# Understanding pseudo-albinism in sole (*Solea senegalensis*): a transcriptomics and metagenomics approach

**DOI:** 10.1038/s41598-019-49501-6

**Published:** 2019-09-20

**Authors:** Patricia I. S. Pinto, Cláudia C. Guerreiro, Rita A. Costa, Juan F. Martinez-Blanch, Carlos Carballo, Francisco M. Codoñer, Manuel Manchado, Deborah M. Power

**Affiliations:** 1 Centre of Marine Sciences (CCMAR), Universidade do Algarve, Campus de Gambelas, 8005-139 Faro, Portugal; 2 LifeSequencing-ADM Nutrition, Parc Cientific Universidad De Valencia, Edif. 2, C/Catedrático Agustín Escardino Benlloch, 9, 46980 Paterna, Spain; 3 Instituto de Investigación y Formación Agraria y Pesquera (IFAPA) Centro El Toruño, Camino Tiro de Pichon s/n, 11500 Cadiz, Spain

**Keywords:** Mechanisms of disease, Bacteria, Transcriptomics, Physiology

## Abstract

Pseudo-albinism is a pigmentation disorder observed in flatfish aquaculture with a complex, multi-factor aetiology. We tested the hypothesis that pigmentation abnormalities are an overt signal of more generalised modifications in tissue structure and function, using as a model the Senegalese sole and two important innate immune barriers, the skin and intestine, and their microbiomes. Stereological analyses in pseudo-albino sole revealed a significantly increased mucous cell number in skin (*P* < 0.001) and a significantly thicker muscle layer and *lamina propria* in gut (*P* < 0.001). RNA-seq transcriptome analysis of the skin and gut identified 573 differentially expressed transcripts (DETs, FDR < 0.05) between pseudo-albino and pigmented soles (one pool/tissue from 4 individuals/phenotype). DETs were mainly linked to pigment production, skin structure and regeneration and smooth muscle contraction. The microbiome (16 S rRNA analysis) was highly diverse in pigmented and pseudo-albino skin but in gut had low complexity and diverged between the two pigmentation phenotypes. Quantitative PCR revealed significantly lower loads of *Mycoplasma* (*P* < 0.05) and *Vibrio* bacteria (*P* < 0.01) in pseudo-albino compared to the control. The study revealed that pseudo-albinism in addition to pigmentation changes was associated with generalised changes in the skin and gut structure and a modification in the gut microbiome.

## Introduction

Flatfish are a fascinating model in which to investigate factors that govern body symmetry and pigmentation patterns in vertebrates. These teleosts become asymmetric during metamorphosis when one of the eyes migrates to the contralateral side of the cranium and the viscera moves in the abdominal cavity to fit to the new flat morphology^1,2^. The morphological changes are accompanied by the establishment of a well-differentiated dorso-ventral pigmentation pattern. In the ocular side, stem chromoblasts differentiate into adult chromatophores (mainly melanophores, xanthophores and iridophores) and larval chromatophores progressively disappear. In contrast, in the blind side, stem cells undergo cytolysis or become iridophores and the skin develops a typical light pigmentation^3^.

The flatfish, in common with other teleosts, dynamically modify their pigmentation pattern in response to short or long-term stimuli such as changes in background colour and substrate, illumination or their physiological state^4^. However, some flatfish can also suffer aberrant and permanent changes in skin pigmentation due to disruption of cellular and molecular mechanisms that govern asymmetric skin remodelling during metamorphosis^5^. The permanent pigmentation abnormalities arising during metamorphosis are known as, i) pseudo-albinism, when a partial or total lack of dark pigmentation occurs on the ocular side of the animal, or ii) ambicolouration, when the blind side of the animal is dark. Pseudo-albinism represents a major challenge for flatfish aquaculture as it transmits a negative perception of the product to consumers and this reduces its market value^2,4^. Moreover, malpigmented animals are not suitable for restocking programs due to the higher predation rates and their reduced survival in the wild^6^.

The flatfish pseudo-albinism disturbance is initiated during a sensitive “pigmentation window” before metamorphosis completion and a range of factors including dietary composition, environmental conditions and modified endocrine responses can disrupt thyroid-mediated metamorphic signalling pathways. High dietary levels of arachidonic acid generate almost complete pseudo-albino fish populations^7,8^, while impaired dietary levels of vitamin A (and retinoid derivatives) or altered thyroid hormone levels (key effectors of metamorphic transformations) also increase abnormal pigmentation rates^2,5,9^. Continuous illumination during pelagic stages may also disrupt the neuroendocrine system through modifying synthesis and secretion of dopamine and α-melanophore-stimulating hormone (MSH)^10^. Currently the effects of pseudo-albinism are considered at the level of the skin, however, the manifestations associated with this syndrome are likely to be more complex. This notion is supported by studies that reveal that skin pigmentary disorders may also be associated with changes in eye translocation and interocular distance^8,11^. In addition, malpigmented fish accumulate an excess of retinal pigment and under high illumination conditions may grow faster than pigmented fish^12,13^. Most research on pseudo-albinism has focused on regulation of the melanogenic biosynthetic pathway in the skin of flatfish larvae^3^, but high-throughput analysis of juvenile stages is likely to shed light on the aetiology and complexity of this condition.

The melanocortin system has been proposed as the main effector of the dorsal-ventral pigmentation pattern in flatfish. The pituitary hormone α-MSH binds to the melanocortin 1 receptor (MC1R) in the skin to activate the downstream regulatory pathways controlling melanophore proliferation and melanin production^4^. This action is antagonized by the paracrine factor agouti-signalling protein (ASIP1) that competes with α-MSH for MC1R binding. ASIP1 is up-regulated in the blind side and in non-pigmented regions of the ocular side in pseudo-albino flatfish and its transitory overexpression induces skin paling and blocks melanogenesis^14^. Although the lack of melanin production in skin is the most evident effect in pseudo-albinism, the disruption of the melanocortin regulatory pathway can also affect feeding behaviour, growth, stress and the immune response^15,16^. This leads us to hypothesize that pseudo-albinism in fish is the visual manifestation of changes in skin but also other tissues and, in the case of those in contact with the environment, may modify their microbial community. In humans, skin pigmentation significantly influenced bacterial diversity^17^ and changes in the skin microbiome have also been observed in various dermatological disorders such as vitiligo, atopic dermatitis and psoriasis e.g.^18,19^. In fish, global microbiome characterization studies are still scarce but are likely to have important implications for aquaculture^20^.

In the present study, to test the hypothesis that pseudo-albinism is the visible manifestation of an underlying shift in animal physiology, transcriptomes (RNA-seq and quantitative PCR) were generated for the skin and intestine of normally pigmented and pseudo-albino juvenile Senegalese sole (*Solea senegalensis*). Histological analysis of the skin and intestine was used to detect modifications in tissue structure. To assess if modified pigmentation in pseudo-albino fish was associated with changes in the microbiome, 16 S rRNA gene amplification coupled to high-throughput sequencing and quantitative PCR validation was performed for the skin and gut in this flatfish species.

## Methods

### Fish, culture conditions and sampling

All procedures were performed in accordance with national and European Union legislation for animal care and experimentation (Directive 86\609\EU) and were authorized by the IFAPA Bioethics and Animal Welfare Committee with registration nr. 26–11–15-374 from the Spanish authorities. Pseudo-albino and normally pigmented 6-month old juvenile sole, belonging to the same spawning batch, were supplied by Cupimar S.A (Cadiz, Spain). As the pseudo-albinism incidence is very low in this species with the current hatchery protocols, the animals were randomly selected from at least 3 different industrial tanks, from a total of 7,500 fish cultivated at a density of 6 kg/m^2^.

To assure aseptic and consistent sampling conditions, fish were transported to the aquaculture station IFAPA-Centro *El Toruño* (minimizing transport time to approx. 30 min) where they were maintained for an acclimation period of ten days so they could recover from handling or transport stress effects. We cannot exclude the possibility that some long-term stress effects might be represented in gene transcripts of pseudo-albino and control fish, but the two groups were always handled and maintained in parallel conditions to minimize differences.

Fish were maintained under natural photoperiod in a single open circuit 400 L tank to neutralize for environmental effects on pseudo-albino fish and their pigmented controls. The tank water renewal rate/day was 5 volumes and water temperature was 20.3 ± 1.0 °C, salinity 35–40 ppt, and fish were fed once daily on commercial dry feed (Skretting, Gemma diamond 1.0 at a 2% biomass rate). All conditions for fish maintenance were comparable to those used at the original aquaculture facilities. No mortality or visible signs of disease were observed during acclimation. N = 9 pseudo-albino and n = 13 pigmented fish (approx. 4 g) were fasted for 24 h and euthanized with 300 ppm MS-222 followed by brain destruction. All fish were rinsed in sterile seawater before dissection, which was carried out under aseptic conditions under a laminar flow cabinet. For histological analysis, a small section of dorsal skin just above the pectoral fin and a section of the anterior intestine were transferred to 4% paraformaldehyde and stored at 4 °C overnight. Individual scales were plucked with forceps from approximately the same dorsal skin region and directly photographed in a microscope attached to a LEICA DFC420 camera under incident light. For microbiome and transcriptome analyses, dorsal skin and anterior gut samples were excised with sterilized dissection material, incubated in RNAlater at 4 °C for 24 h and stored at −20 °C until DNA/RNA extraction.

### Histological analyses

Individual skin and gut samples from n = 5–6 pigmented and n = 4–6 pseudo-albino juvenile sole were fixed overnight at 4 °C in 4% paraformaldehyde, washed in PBS and stored in 70% ethanol. Skin samples were decalcified overnight in 0.5 M ethylenediaminetetraacetic acid (EDTA), pH 8 prior to processing. Skin and gut samples were dehydrated in a graded ethanol series (70–100%), saturated in xylene and impregnated with paraffin wax. Serial 5 μm sections were mounted on poly-L-lysine coated glass slides and stained with haematoxylin and eosin (H&E) to evaluate general tissue organization. Masson’s Trichrome staining was performed to differentiate collagen, muscle and mineralized layers. Stained sections were analysed using a DM2000 microscope coupled to a DFC480 digital camera (Leica). Image J-1.51k (http://rsbweb.nih.gov/ij/) was used to estimate the number of mucous cells in the epithelia spanning 3 scales, every 4th section (15 μm) of skin in 4 sections/individual. In the gut, the abundance of mucous cells, the muscle layer thickness and the *lamina propria* thickness in three different positions (crypt, mid-point and tip) of 4 *villi* per section was determined in 3–4 sections/individual.

### DNA and RNA extractions

DNA for microbiome analysis was extracted from the dorsal skin and anterior gut using a DNeasy Blood & Tissue Kit (Qiagen). Modifications in the manufacturer’s protocol included pre-digestion with lysozyme, RNAse treatment and mechanical disruption with 0.1 mm zirconia/silica beads (Biospec) using a Bertin Precellys 24 homogeniser (20 s at 6,800 rpm). Total RNA for RNA-seq was extracted using an E.Z.N.A. Total RNA Kit I (Omega-Biotek) following mechanical disruption of approx. 30 mg of skin and 10–30 mg of gut using 5 mm iron beads (Qiagen) for 2 × 20 sec at 6,800 rpm in a Precellys 24. RNA samples were treated with DNAse to remove possible DNA contamination. DNA/RNA quality and integrity were analysed using a Nanodrop spectrophotometer and 1% agarose gel electrophoresis.

### Transcriptomics: RNA-seq library preparation, sequencing and data analysis

Four RNA-seq libraries were constructed using pools of total RNA from dorsal skin and anterior gut of pseudo-albino or pigmented sole (n = 4 individuals/pool), using individuals selected to have a similar weight and a total lack of dark pigmentation, in the case of pseudo-albino fish. A Bioanalyser confirmed all extracted RNAs had an integrity values (RIN) > 7.2. Equal quantities (250 ng) of RNA from each specimen were pooled per group and used to prepare RNA-seq libraries (designated SkinP and GutP for pigmented fish and SkinA and GutA for pseudo-albinos), using an Illumina TruSeq RNA sample preparation kit and TruSeq index adaptors. Average fragment size (260 bp) and purified library concentration (24–30 nM) were determined using a Bioanalyser prior to sequencing at Lifesequencing S.L.-ADM (Valencia, Spain) using an Illumina NextSeq. 500 platform.

Library sequencing generated 526 × 10^6^ of 75 bp single-end (SE) raw reads. These were cleaned using SeqTrimNext^21^ and mapped onto the *Solea senegalensis* representative transcriptome v4.1 using Bowtie^22,23^. Differential expression was assessed using RobiNA/EdgeR^24^ with pairwise comparisons between tissues (skin *vs* gut) or pigmentation (pigmented *vs* pseudo-albino), using a false discovery rate (FDR) < 0.05. Stand-alone Blast (E-value < 10^−10^) of all differentially expressed transcripts identified their Ensembl zebrafish orthologues (GRCz/10GCA000002035.3 assembly, https://www.ensembl.org/). Functional analysis was run with the Cytoscape-ClueGO plug-in^25^ using as the input zebrafish orthologues for each differentially expressed transcript of Senegalese sole. Enrichment analyses (right-sided hypergeometric test) were run selecting the gene ontology Biological Process (GO-BP) terms for zebrafish (13/05/2017) between levels 3–8. Terms were considered significantly enriched at an FDR (Benjamini–Hochberg) *P* < 0.05 and minimum of three genes/4% of the GO-BP genes represented in the list. Enriched GO terms were grouped into functionally related networks using an initial group size of 1, a group merging setting of 50% and a Kappa score of 0.4.

### Confirming transcript differential expression by quantitative PCR (qPCR)

The relative standard curve method and EvaGreen chemistry were used, as previously described^26^. Duplicate reactions contained 300 nM of each specific primer (Supplementary Table S1) and 2 μL of diluted (1:5) cDNA (n = 5 individuals/group). Efficiencies of standard curves (serial dilutions of quantified amplicons) ranged between 85–105% with R^2^ > 0.98. The stability of two reference genes (*gapdh2* and *ubq*, previously used in *Solea senegalensis*^27^) was evaluated by analysing their Cts (threshold cycles) in both tissue panels using RefFinder (150.216.56.64/referencegene.php). Since both reference genes were stable, their geometric mean was used to normalize candidate gene expression, which was expressed relative to the average of the pigmented sole group (considered the control condition).

### Metagenomics: 16 S rRNA gene microbiome library preparation, sequencing and data analysis

Two libraries per condition (pigmented or pseudo-albino) were constructed for the dorsal skin and for the anterior gut, using two pools of DNA from 2 individuals each (also used for RNA-seq). One DNA sample of the tank water and food were also analysed. Sequenced libraries were designated SkinP1, SkinP2, GutP1 and GutP2 for pigmented fish; SkinA1, SkinA2, GutA1 and GutA3 for pseudo-albino fish; H_2_O for tank water and Food for the food pellet samples. Library preparation followed the 16 S Metagenomic Sequencing Library Preparation protocol for the Illumina MiSeq system, using optimized primers^28^ targeting the hypervariable V3 and V4 regions of the 16 S rRNA gene. Libraries were sequenced on an Illumina MiSeq instrument at Lifesequencing S.L.-ADM. Raw reads were cleaned, merged into paired-end reads, chimeras cleaned and sequences used for identification and classification of operational taxonomic units (OTU) as previously described^29^, using comparisons against the NCBI 16 S rRNA database. A pipeline developed by Lifesequencing S.L.-ADM was used to obtain hierarchy clustering and conduct principal component analyses using the R Packages ggplot2, pheatmap and ggbiplot^29^.

### Confirming the presence of bacterial genera using RT-PCR and qPCR

RT-PCRs for microbiome result validation were carried out using specific primers optimized for the detection of *Vibrio* genus members (degenerate primers for recombinase A, 689 bp amplicon^30^) and for the *Mycoplasma* genus (primers for the 16 S rRNA gene, amplifying 1013bp^31^)- Supplementary Table S1. *Vibrio*/*Mycoplasma* amplification was established for anterior gut DNAs from 9 pseudo-albinos and 13 pigmented juvenile sole; the individual samples used for the microbiome library production (n = 4/group) were also included in this analysis. These amplicons were cloned into pGem-T easy (Promega) and 8–10 clones/group were sequenced to confirm genus identity and infer species-specific amplification. In addition, *Vibrio* or *Mycoplasma* genus-specific primers targeting a smaller region of the 16 S rRNA gene^32,33^ (Supplementary Table S1) were optimized and used for the qPCR quantification of these bacteria in the pseudo-albino or pigmented sole gut DNAs. qPCR reactions were performed as indicated above, copy number of *Vibrio* or *Mycoplasma* were calculated as previously described^34^ and then normalized in relation to microgram of total DNA extracted from the anterior gut.

### Statistical analysis

Stereological measurements, qPCR of *Vibrio*/*Mycoplasma* bacteria in sole gut and the expression of selected differential transcripts in sole skin and gut are represented in bar charts with the mean ± SEM (standard error of the mean) of the results. Significant differences between the groups were evaluated by one-way analysis of variance (one-way ANOVA) in SigmaStat v.3.50 (Systat) Software using log2 transformed data, followed by a Tukey test. Pearson correlation analysis was used to test the relationship between log2 specific gene expression levels obtained by RNA-seq and gene transcript abundance measured by qPCR. Significance levels were set at *P* < 0.05.

## Results

### Pseudo-albino fish have structural differences in skin and the gut

To assess the long-lasting morphological, microbial and expression patterns that occur in pseudo-albino juvenile soles, specimens cultivated under the same conditions for six months were randomly chosen from the rearing tank (pseudo-albinism incidence lower than 0.08%). No significant differences in weight were observed between the randomly selected pigmented (3.9 ± 0.5 g, n = 4) and pseudo-albino (3.7 ± 0.4 g, n = 4) fish, which were sampled under the same conditions.

**Figure 1:**
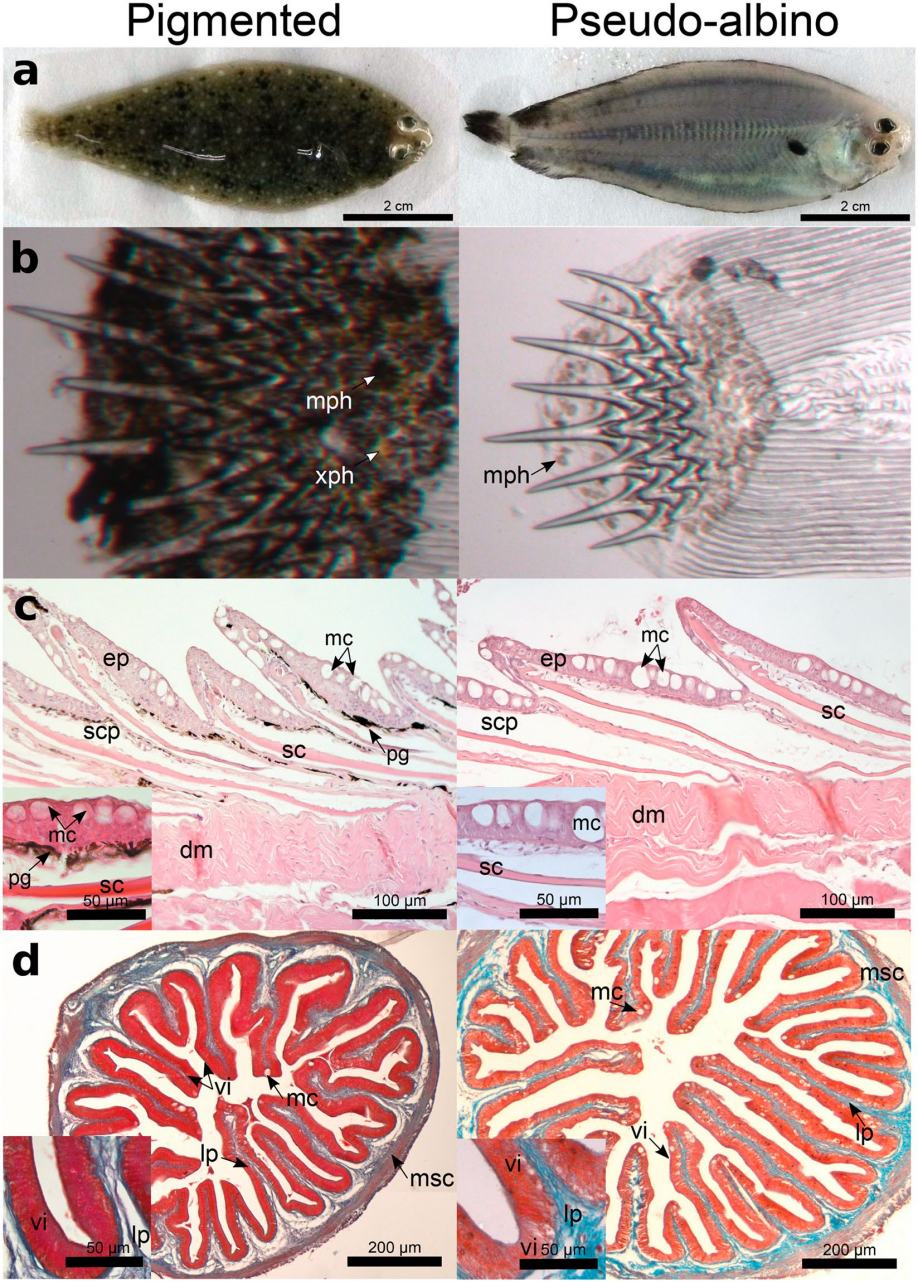
External and internal morphology of pigmented and pseudo-albino juvenile sole. (**a**) Pigmented and pseudo-albino juvenile sole photographed from the ocular side demonstrating clear differences in pigmentation patterns. (**b**) Microscopic observation of skin and scales sampled from the mid-region of the dorsal skin identifying dendritic melanophores (mph, black/brown) and xanthophores (xph, yellow) organized in dense patches, in pigmented fish, and non-dispersed round melanophores in the pseudo-albino fish. (**c**) Haematoxylin & Eosin stained longitudinal histological sections of the dorsal skin demonstrating the presence of pigment (pg) in the upper dermis of pigmented but not pseudo-albino sole. (**d**) Masson’s trichrome stained histological sections of the anterior gut illustrating the thicker muscle wall (msc) in the pseudo-albino fish relative to the pigmented fish. ep: epidermis, dm: dermis, scp: scale pocket, sc: scale, mc: mucous cells, vi: *villi*, lp: *lamina propria*, *msc: muscle*.

Observation of the external morphology of the pigmented sole confirmed their characteristic dark patchy pigmentation on the dorsal, ocular skin relative to the lack of pigmentation in pseudo-albino fish (Fig. 1a). No major differences between groups were observed in the ventral, blind-side (not shown), which lacked pigmentation in both groups. Microscopic observation of the skin in pigmented fish revealed the typical arrangement of melanophores and xanthophores (Fig. 1b). This pattern was almost completely absent in pseudo-albino fish, which had a very reduced number of small round melanophores that had lost their typical dendritic shape.

Histological analysis of pigmented dorsal skin revealed the scales localized in individual scale pockets inserted in the dermis and covered by a mucous cell-rich epidermis underlaid by numerous melanophores in the pigmented fish (Fig. 1c, see inset). The structure of skin in pseudo-albino fish was similar to pigmented skin but few melanophores were evident. The histology of the gut revealed the circular and longitudinal muscles, the *lamina propria*, the *villi* covered by enterocytes and some goblet (mucus-producing) cells (Fig. 1d). Scarce pigmentation was found in the outer serosa of both pigmented and pseudo-albino fish.

**Figure 2:**
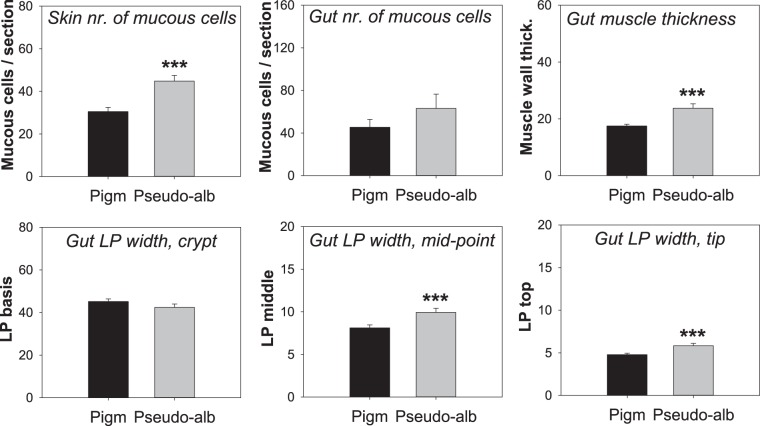
Stereological measurements in pigmented and pseudo-albino juvenile sole. Bars represent the mean ± SEM of mucous cell number in the dorsal skin or anterior gut of n = 5–6 pigmented and n = 4–6 pseudo-albino juvenile sole (4 sections per individual). In the gut, the thickness of the muscle wall layer and the width of the *lamina propria* were measured in three different positions of the villi, in µm (crypt, mid-point and tip; 4 villi per section, 3–4 sections per individual). Symbols above the bars denote significant differences between pseudo-albino sole and the pigmented control identified using one-way ANOVA: *** for *P* < 0.001.

Stereological measurements of the skin and gut revealed a significantly higher number of mucous cells in pseudo-albinos relative to pigmented fish (*P* < 0.001 in the skin; Fig. 2), while in the gut this difference was not statistically significant. The main differences between the gut in pseudo-albino and pigmented sole was the significantly thicker muscle wall (*P* < 0.001) and thicker *lamina propria* at the mid-point and tip of the *villi* (*P* < 0.001) in pseudo-albino fish.

### Skin and gut transcriptomes differ between pseudo-albino and pigmented sole

A similar read number was obtained for the four RNA-seq libraries generated from the dorsal skin and anterior gut of pigmented or pseudo-albino juvenile sole (Supplementary Table S2) and the sequencing depth was 8.0 Gb/library. Bioinformatics analyses identified 91% of useful (filtered) reads, 82% of which mapped to the *S*. *senegalensis* representative transcriptome^23^.

**Figure 3:**
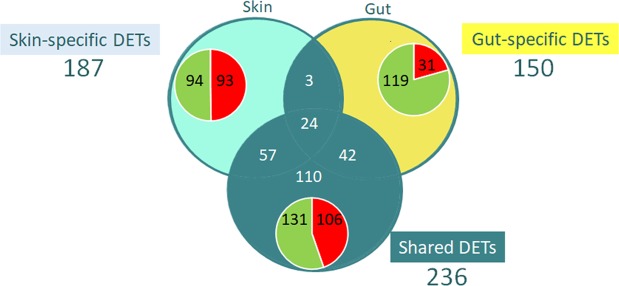
Differentially expressed transcripts between pigmented and pseudo-albino sole. Venn diagrams show the number of candidate differentially expressed transcripts (DETs, identified at FDR < 0.05) between pseudo-albino and pigmented fish. These include DETs only detected in skin (“skin-specific”), DETs only in the gut (“gut-specific”) or “shared” transcripts differentially expressed irrespective of the tissue. Inset pie charts show the proportion of up- (higher expression in pseudo-albino than pigmented) or down-regulated (pigmented > pseudo- albino) transcripts within each subgroup.

Comparison of the transcripts expressed in skin and gut, irrespective of pigmentation, identified 25,997 candidate differentially expressed transcripts (DETs). Tissue-specific comparisons between pigmented and pseudo-albino fish identified 573 DETs and 271 were differentially expressed in the skin and 219 in the gut. When a global comparison of transcripts between the two groups was carried out irrespective of the tissue, 233 “shared DETs” were identified (Fig. 3, Supplementary Table S3). Comparison of DET identities listed 187 unique DETs specifically identified for skin, 150 unique DETs specific for the gut and 236 “shared DETs”. Supplementary Table S4 lists the expression and annotation data for the 573 identified candidate DETs.

**Figure 4:**
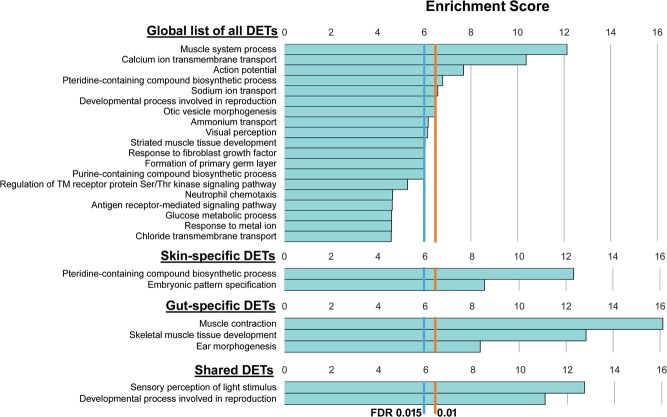
Functional enrichment analyses. Bar graphs show the main functionally related networks (groups) of Biological Processes that were overrepresented in the global list of 573 candidate DETs between pigmented and pseudo-albino fish, and in the tissue specific or shared DETs lists. Enrichment for gene ontology (GO) terms was carried out using the ClueGO plugin and Cytoscape software with the minimum significance set at 0.05 FDR. Represented groups had a significant enrichment (FDR < 0.05) and associated related significant GO terms according to their functional classification. Each group is labelled after its most significant term. Bar length corresponds to the significance of each group in the ClueGO network output measured by enrichment score (−log2 (group FDR). Vertical lines show significance thresholds of higher stringency: FDR 0.015 (enrichment score = 6) or FDR 0.01 (enrichment score 6.6). For detailed lists of all significantly enriched GO terms and groups consult Supplementary Tables S5 and S6.

To gain insight into the main molecular pathways where differences in expression were found between the skin or gut of pseudo-albino in relation to pigmented fish, global gene ontology (GO) analyses were run using the list of 573 DETs and identified 82 significantly enriched biological processes (FDR < 0.05; Supplementary Table S5). These were mainly related to muscle contraction, pigmentation regulatory pathways, hormone regulation, ion transport and tissue morphogenesis (Fig. 4). Categories associated with pigment biosynthesis and cell differentiation and fate were among the most differentially expressed in skin while in the gut, muscle-related categories were most represented (Fig. 4 and Supplementary Table S6). Finally, DETs shared between the two tissues were mainly enriched in GO categories related to sensory perception of light stimulus and to hormonal regulation.

### Quantitative PCR validation confirmed DETs identified in RNA-seq data

A focused analysis of the candidate DETs identified several genes specifically related to pigmentation in the skin (see Table 1). Moreover, DETs were also found that were related to the retinoic acid signalling pathway; hormonal regulation of reproduction, feed intake and energy metabolism; gas transport and acid/base regulation; mucin production; immune system; transcription factors involved in epithelial cell differentiation; extracellular matrix composition and muscle contraction.

**Table 1:** Differentially expressed transcripts (DETs) from selected categories.

Category	Transcript annotation	Gene name	Acc number	Global	skin	gut
Pigmentation	Melanocortin 1 receptor	*mc1r* ^#^	unigene100575	−3.1	−3.1	ns
	Melanocortin-5 receptor	*mc5r*	unigene18004	ns	−9.0	ns
	Agouti signalling protein 2	asip2*	unigene59061	5.6	6.4	ns
	Muscarinic acetylcholine receptor M2	*chrm2*	unigene49052	−2.8	ns	ns
	Adrenoceptor alpha 1D	*adra1d*	unigene16344	ns	6.6	ns
	Melanoregulin	*mreg/dsu*	unigene7025	−3.3	−3.4	ns
	Stathmin-4	*stmn4*	unigene39977	ns	−3.7	ns
	GTP cyclohydrolase 1	*gch1* ^*#*^ *	unigene584207	ns	−9.0	ns
	Sepiapterin reductase	sprb*	unigene45253	ns	−5.2	ns
	Cytosolic purine 5′-nucleotidase	nt5c2*	unigene23390	2.3	2.5	ns
	Purine nucleoside phosphorylase	*pnp*	unigene33375	ns	−2.4	ns
	Adenosine deaminase domain-containing protein 2	*adad2*	unigene55885	ns	3.0	ns
	Hypoxanthine-guanine phosphoribosyltransferase	*hprt1*	unigene34021	−2.7	−2.8	ns
Hormonal regulation	Cocaine- and amphetamine-regulated transcript 2a	*cart2a*	unigene1742	−3.4	−2.9	−5.6
	Urocortin-3	*ucn3*	unigene246776	−3.3	ns	−6.2
	Apelin receptor A	*aplnra*	unigene692623	ns	ns	−4.0
	Steroid 17-alpha-hydroxylase/17,20 lyase	*cyp17a1*	unigene602139	2.8	3.0	ns
	Myostatin-2	*mstn2*	unigene653244	ns	6.8	ns
	Thyroid hormone receptor alpha A	thraa*	unigene12340	−2.8	ns	ns
	Somatostatin-2	sst2*	unigene441354	ns	ns	−3.9
	Insulin	ins*	unigene415183	−7.6	ns	−7.6
Retinoic acid pathway	Retinol dehydrogenase 11	*rdh11*	unigene95290	−2.5	−2.8	ns
	All-trans-retinol 13,14-reductase	*retsat*	unigene102700	−3.5	−4.1	ns
	Aldehyde dehydrogenase 1 family member A3	aldh1a3*	unigene16433	ns	ns	3.0
	Retinoic acid receptor responder protein 3	*rarres3*	unigene69004	ns	−6.0	ns
	RPE-retinal G protein-coupled receptor	*rgr*	unigene4613	−2.4	−2.7	ns
Transcription factors	Homeobox C13	hoxc13a*	unigene33250	ns	6.9	ns
	Homeobox protein Hox-C12	*hoxC12a*	unigene32210	ns	3.2	ns
	SRY-related HMG-box protein 3	sox3*	unigene31361	ns	3.8	ns
	Fibroblast growth factor 3	*fgf3*	unigene448254	ns	5.8	ns
	Fibroblast growth factor 23	*fgf23*	unigene114645	3.4	5.4	ns
	LIM/homeobox protein Lhx1	*lhx1*	unigene78221	2.8	5.7	ns
	Forkhead box protein D5	*foxd5*	unigene228150	ns	−3.9	ns
	Homeobox B1 b	*hoxb1b*	unigene227560	ns	−5.7	ns
	Homeobox protein Hox-B6b	*hoxb6b*	unigene31921	ns	−5.7	ns
	Fibroblast growth factor 4	*fgf4*	unigene163077	−3.9	−3.5	ns
	SRY-box 5	*sox5*	unigene230814	ns	−5.8	ns
	Homeobox protein Hox-D4b	*hoxd4b*	unigene47059	ns	ns	−3.1
	SRY-box 10	*sox10*	unigene238791	ns	ns	−4.0
	Msh homeobox 1	msx1*	unigene11948	ns	ns	−6.7
Gas transport and acid-base regulation	Hemoglobin subunit alpha-1	*hbaa1* ^#^	unigene36770	4.2	6.4	ns
	Hemoglobin cathodic beta chain	*hbba2* ^#^	unigene689117	4.2	5.3	ns
	Hemoglobin beta embryonic-1.1	*hbbe1*.*1*	unigene44588	ns	6.1	ns
	Erythropoietin receptor	*epor*	unigene417097	3.2	4.0	ns
	DCC netrin 1 receptor	*dcc*	unigene240403	5.1	4.4	6.1
	T-cell acute lymphocytic leukemia protein 1	*tal1*	unigene574299	ns	6.0	ns
	Carbonic anhydrase 4	*ca4a* ^#^	unigene11571	ns	−3.1	−5.6
	D-beta-hydroxybutyrate dehydrogenase	*bdh1*	unigene33612	ns	−4.3	ns
Mucin production	Polypeptide N-acetylgalactosaminyltransferase-protein 17	*galnt17* ^#^ *	unigene421602	3.2	ns	6.4
	Mucin-5AC-like	*muc5acl*	unigene63498	ns	3.4	ns
	Mucin-2-like	*muc2l*	unigene512244	ns	−4.3	ns
	Mucin-3A-like	*muc3al*	unigene585886	ns	ns	−5.9
	Mucin 2 (intestinal)	muc2i*	Unigene52046	ns	ns	−2.7
	Mucin 13 cell surface associated	muc13*	unigene21876	ns	−2.4	ns
Immune system	C-C chemokine receptor type 3	*ccr3*	unigene144291	3.6	3.5	ns
	Complement C3	*c3*	unigene553179	ns	6.1	ns
	Complement C1q-like adipose specific protein	*c1q* ^#^	unigene620235	11	6.8	11
	Complement C1r-A	*c1ra*	unigene22322	ns	4.0	ns
	Complement factor H	*cfh* ^#^	unigene94769	5.7	5.3	5.9
	Ladderlectin	*ll* ^*#*^	unigene326025	ns	−3.2	ns
	Arginase-1	*arg1*	unigene44687	ns	ns	−5.5
	Fish-egg lectin	*fel*	unigene92796	ns	ns	−4.0
	C-C motif chemokine ligand 28	*ccl28*	unigene521206	ns	ns	−4.1
	Interleukin 1 beta	il1b*	unigene346347	ns	ns	−5.9
	Myeloperoxidase/Eosinophil peroxidase-like	mpx*	unigene23826	−2.7	ns	−3.5
	C-C motif chemokine 20	*ccl20*	unigene78524	−2.6	ns	−2.9
	Interleukin 8-like	*il8*	unigene477191	2.4	ns	2.6
Extracellular matrix	Keratin, type I cytoskeletal 18	*krt18*	unigene325393	ns	−2.9	ns
	Fibromodulin-like/extracellular matrix protein 2-like	*fmod/ecm2*	unigene190864	ns	−3.4	ns
	Testican-1	*spock1*	unigene1560	ns	−3.8	ns
	Sulfate glucosamine 3-O-sulfotransferase 2	*hs3st2*	unigene44891	3.0	ns	5.9
	Zona pellucida sperm-binding protein 3	zp3a2*	unigene51388	2.6	ns	4.0
	Hyaluronan-binding protein 4	*habp4* ^#^	unigene292551	3.1	ns	3.7
	Protocadherin-11 × -linked	*pcdh11X*	unigene45384	ns	ns	3.7
	Collagen alpha-1(XI) chain-	*col11a1*	unigene327347	ns	ns	5.9
	Laminin subunit beta-4	*lamb4*	unigene126475	ns	ns	−5.8
Muscle contraction	Myosin, heavy polypeptide 2, fast muscle-specific	*myhz2*	unigene442827	ns	ns	−5.7
	Myosin-7	*myh7* ^*#*^ *	unigene69294	ns	ns	−4.6
	Myosin, heavy polypeptide 1.1, skeletal muscle-like	*myhl*	unigene572942	ns	ns	−2.7
	Myosin light chain, phosphorylatable, fast skeletal muscle	*mylpf* ^*#*^	unigene440524	ns	ns	−3.8
	Troponin I, slow skeletal muscle	*tnni1d*	unigene19640	ns	ns	−3.1
	Fast muscle troponin T isoform	*tnnt3b*	unigene429220	ns	ns	−3.5
	Troponin i4b, tandem duplicate 1	*tnni4b*.*1*	unigene584366	ns	ns	−3.5
	Myosin, light polypeptide 3, skeletal muscle	*mylz3*	unigene440524	ns	ns	−3.8
	Myosin, light polypeptide 2b, regulatory, cardiac, slow	*myl2*	unigene29268	ns	ns	−3.8
	Fast skeletal muscle troponin C	*tnnc2*	unigene280197	ns	ns	−4.2
	Myosin heavy chain, fast skeletal muscle-like	*myha*	unigene429931	ns	ns	−5.6
	Tropomyosin 3	*tpm3*	unigene588468	ns	ns	−7.3

Presented are the transcript annotation name, gene symbol (following the nomenclature from www.zfin.org whenever zebra fish homologs were found), Unigene transcript accession numbers (from SoleaDB) and the log2 of fold changes for significant differentially expressed transcripts (DETs) between the skin (one pool/phenotype) and gut (one pool/phenotype) libraries of pseudo-albino and control sole or using a global statistical analysis to identify DETs irrespective of the tissue. ns = non-significant. ^#^ indicates genes for which more than one transcript was found to be significantly regulated; the DET with highest fold change is presented and other DETs are reported in Supplementary table S4. * indicates genes from selected categories confirmed by quantitative PCR (for the complete list of genes and tissues analysed see Supplementary Table S1).

**Figure 5:**
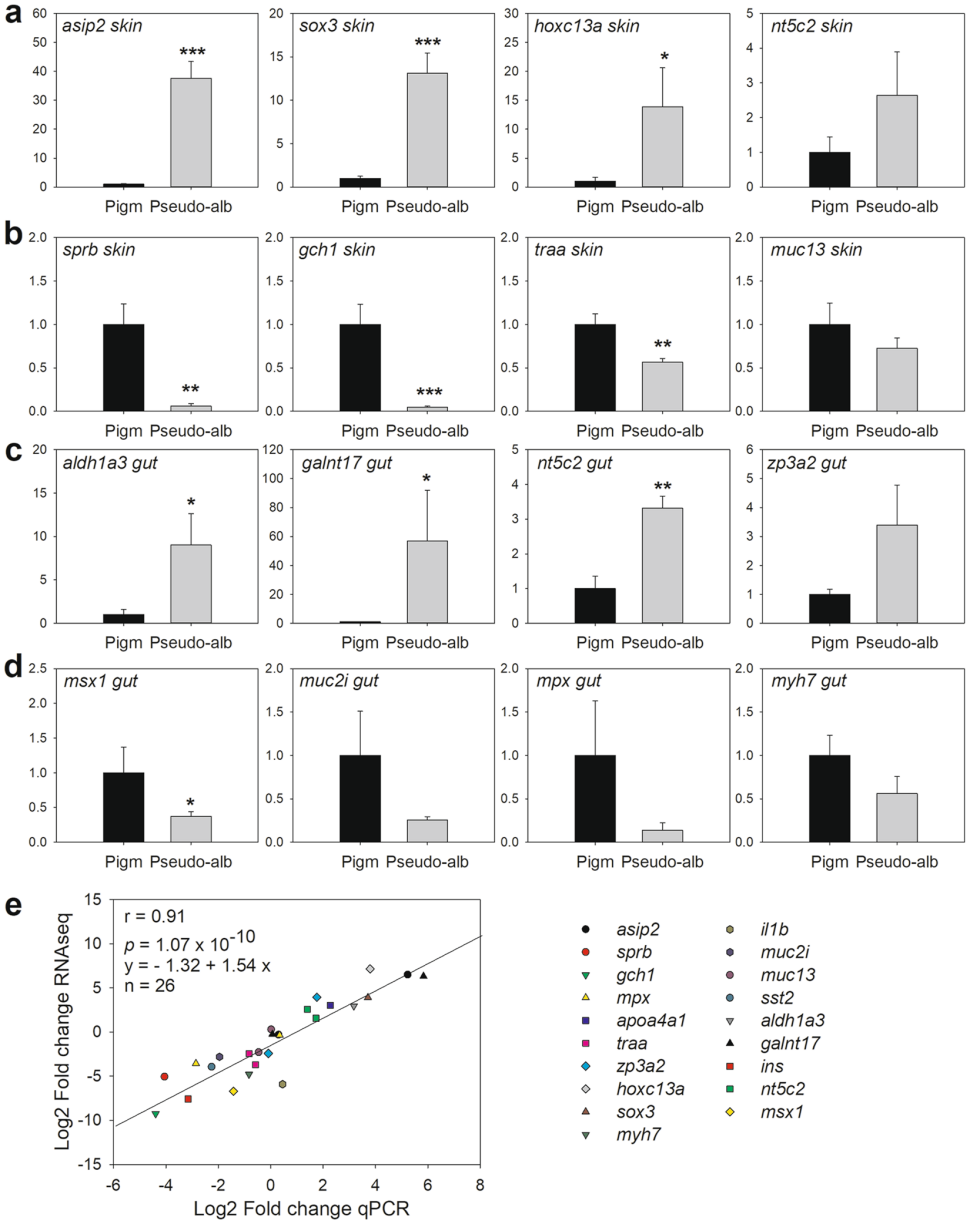
Confirmation of DETs by quantitative PCR (qPCR). The expression of candidate DETs detected to be up- or down-regulated in skin (panels a and b) or up- or down-regulated in gut (**c**,**d**) in RNA-seq was quantified in 5 pigmented (Pigm, black bars) and 5 pseudo-albino (Pseudo-alb, grey bars) sole. Results are represented as the mean ± SEM of the relative expression quantified by qPCR and normalized using the geometric mean of the reference genes *gapdh2* and *ubq* and expressed relative to the pigmented control condition. Symbols above the bars indicate significant differences between pseudo-albino sole and the pigmented control identified by one-way ANOVA: * for *P* < 0.05, **P < 0.01 and ***P < 0.001). Panel e shows the correlation between fold-changes of transcript expression measured between pigmented *vs* pseudo-albino fish by RNA-seq or qPCR, both log2 transformed. Pearson correlation coefficient and probability (r and p) are presented as well as the equation of the linear regression and number of points analysed. Transcript name abbreviations and accession numbers can be found in Supplementary Table S1. Some transcripts from the “shared DET” group were quantified in both tissues; all quantified expression results are included in panel E while for A-D the tissue with higher differences was selected for representation.

Expression of nineteen selected DETs (Supplementary Table S1, Table 1) was evaluated by qPCR using 5 individual samples from pigmented or pseudo-albino sole (Fig. 5). Significant up- or down-regulation was confirmed for six genes in pseudo-albino skin (*asip2*, *sox3*, *hoxc13a*, *sprb*, *gch1*, *thraa*). High individual variation was obtained for the gut samples, with 4 out of the tested transcripts passing statistical significance (*aldh1a3*, *galnt17*, *nt5c2* and *msx1*). Nevertheless, a highly significant, positive correlation, was found between the qPCR and RNA-seq analyses (r = 0.91, *P* = 1.07 × 10^−10^; Fig. 5e), which supported an overall concordance between the results of the two techniques despite the low replicate number for RNA-seq analysis.

### Bacterial microbiomes differ between pigmented and pseudo-albino juvenile sole in the gut but not in the skin

Tissue or phenotype differences in the diversity of bacterial populations present in juvenile sole dorsal skin and anterior gut were evaluated using 16 S rRNA gene high-throughput sequencing. Approx. 1.7 million sequences were produced from two replicate libraries per tissue/phenotype and one sample for the food or environmental water. An average of 57,644 good-quality reads per library were generated (Supplementary Table S7) and rarefaction curves showed all libraries were close to saturation at the obtained sequencing depth, indicating most bacterial diversity in the samples was captured (Supplementary Fig. S1).

The water microbiome appeared to have the highest diversity followed by the food and the skin, while the gut microbiome had the lowest diversity and the least OTUs (operational taxonomic units) detected at both genus and species levels. This agrees with the average Shannon diversity indexes (SHI) obtained (Supplementary Table S9).

**Figure 6:**
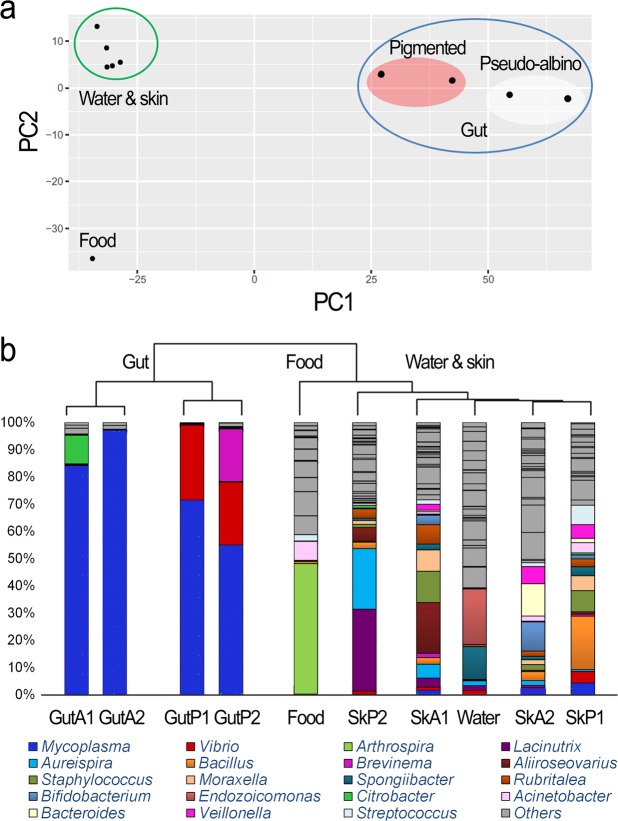
Composition and grouping of microbiomes. (**a**) Principal component analysis (PcoA) separating skin and water microbiome samples from food and gut along components PC1 and PC2, with evident separation between pigmented and pseudo-albino gut samples. (**b**) Hierarchal clustering tree that separated the gut from food, water and skin microbiomes. The diversity within each individual library is represented by stacked bars with colours indicating the main detected genera, with n = 1 library for water or food and n = 2 microbiome libraries/group for skin or gut. Each of the analysed microbiome libraries represented a pool of DNA from 2 individuals. A total of 4 pigmented (P) sole or 4 pseudo-albino (A) sole were used to generate 2 libraries per analysed group.

Principal component analysis (PCoA) and clustering analysis grouped the skin microbiomes together with the water, with no evident separation between pigmentation phenotypes (Fig. 6a,b). A separation was found between the skin cluster and the gut microbiomes, which also separated pigmented and pseudo-albino fish. Supplementary Tables S8 and S9 list, respectively, the main detected bacterial genera or species. Eighteen genera were detected at >1% in water, the most abundant being *Endozoicomonas*, *Spongiibacter* and *Marinobacter*, comprising 25% of the tank water microbiome; all belonged to the *Proteobacteria* phylum that covered 70% of detected OTUs in water (Supplementary Table S8). The food pellet microbiome was dominated by the cyanobacteria *Arthrospira*, which accounted for 40% of the detected bacteria, followed by 12 other genera belonging to the *Proteobacteria* or *Firmicutes* phyla.

In the skin, a high number of genera (49) were detected at >1% but each represented a low proportion (1.7% in average) of the total microbiome (Fig. 6 and Supplementary Table S8). The skin microbiomes were diverse, did not cluster by skin phenotype and appeared to be characteristic of each of the independent samples included in the analysis. In contrast, the gut microbiomes were composed of four main genera (Fig. 6 and Supplementary Table S8) and were dominated by *Mycoplasma* (composing 56–71% of the microbiome in pigmented fish and 87–100% in pseudo-albino). The *Vibrio* genera was highly represented in the gut of pigmented fish (23–27%) and was a small proportion (0.2%) of the gut microbiota in the pseudo-albino fish. The main species detected by 16 S library sequencing were *Mycoplasma muris*, *M*. *microti*, *Vibrio parahaemolyticus*, *V*. *atypicus* and *V*. *scophthalmi* (Supplementary Table S9).

### Confirmation of the main bacterial genera and abundance in pigmented and pseudo-albino sole gut

**Figure 7:**
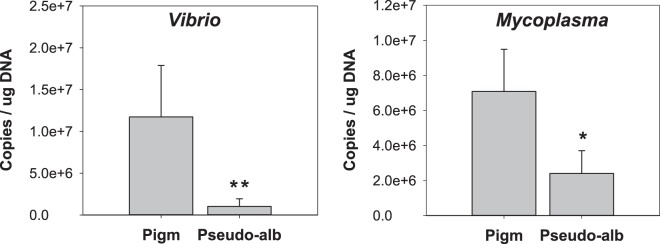
Quantification of *Vibrio* and *Mycoplasma* bacteria in the gut of pigmented and pseudo-albino juvenile sole. Bars represent the mean ± SEM for the quantification of bacteria from the *Vibrio* or *Mycoplasma* genera, using genus-specific primers (Supplementary Table S1) and DNAs from the gut of n = 13 pigmented and n = 9 pseudo-albino juvenile sole, normalized by the µg of DNA used in each PCR. ** and * indicate significant differences compared to the control with *P* < 0.01 or *P* < 0.05, respectively, evaluated by one-way ANOVA.

Two approaches were taken to validate the gut microbiome from pigmented and pseudo-albino sole (obtained by 16 S sequencing from n = 4 animals per group). Using a larger population of juvenile sole, RT-PCRs with genus-specific primers confirmed the high representation of *Vibrio* bacteria in the gut of the pigmented fish analysed (8/13) and low representation in the gut of the pseudo-albino fish analysed (1/9). *Mycoplasma* were detected in all pigmented fish (13/13) and in a lower proportion of pseudo-albino fish (6/9). Cloning and sequencing confirmed the microbiome results at the species level: the *Vibrio* clones from pigmented fish matched *V*. *scophthalmi* (7 clones) or the closely related species *V*. *ichthyoenteri* (1 clone); *Mycoplasma* clones from pigmented sole matched *M*. *muris* (2/8) or *M*. *microti* (6/8), while all 10 *Mycoplasma* clones from the pseudo-albino gut matched *M*. *microti*. Genus-specific primers designed for qPCR confirmed the significantly lower load of both *Vibrio* (p < 0.01) and *Mycoplasma* (p < 0.05) in the gut of pseudo-albino sole compared to the normally pigmented fish (Fig. 7).

## Discussion

Pseudo-albinism is a pigmentation disorder observed in flatfish aquaculture, which depreciates the commercial value of affected fish. Unlike flounder or turbot, pseudo-albinism scarcely occurs in sole aquaculture due to the species fast development until settlement and the short duration of the pigmentation sensitive window^1^. In spite of the low incidence of pseudo-albinism in the cultured population, it can be easily be induced in sole larvae by supplying high levels of dietary arachidonic acid during premetamorphosis^3,7,11^, making the sole an excellent model to investigate the molecular basis of pigmentation anomalies.

Previous research in pseudo-albino post-larvae revealed that adult chromatophore differentiation was blocked after metamorphosis and was associated with round-shaped melanophores and xanthophores that disintegrate at a higher rate^2^. In this study the pseudo-albino juveniles lacked visible xanthophores and only rounded melanophores were observed in skin. Other associated changes were an increase in skin goblet (mucous) cell number and in the gut a thicker muscle layer and *lamina propria*. These findings indicate that in addition to the pigmentation disorder, pseudo-albinism is associated with other structural modifications as previously suggested^8,11^.

Pigmentation in fish results from a balance between the adaptive “physiological” changes in response to environmental stimuli and the long lasting “morphological changes” that affect pigment synthesis and chromatophore abundance^4^. Expression data from a previous study of pseudo-albino post-larvae suggested that *asip1* and *slc24a5* may be the major effectors leading to defects in melanin production^2^. Our skin transcriptome of pseudo-albino juvenile sole suggested a dysregulation of melanocortin-agouti signalling, pigment biosynthesis and pigment dispersion pathways, suggesting a global disruption of chromatophore dynamics in the skin. The pituitary hormone α-MSH is a key regulator of fish pigmentation and stimulates proliferation, pigment production and dispersion when it binds to its receptors MC1R and MC5R in skin melanophores and MC5R in xanthophores^4,35^. In our study in pseudo-albino sole skin, *mc1r* and *mc5r* (previously shown to be overexpressed in hypermelanosis in Japanese flounder^36^) were down-regulated, supporting their involvement in permanent changes of skin pigmentation in fish and corroborating the utility of our RNA-seq results despite the low sample number used.

ASIP1 antagonizes the effects of α-MSH, which via melanocortin receptors inhibits melanoblast differentiation and stimulates iridophore proliferation, and overexpression of *asip1* has been proposed as the main cause of sole pseudo-albinism^2,14^. In the present study, the paralog *asip2* (unigene59061; Supplementary Fig. S2 and Fig. 5a) rather than *asip1* (unigene2880, not shown) was highly up-regulated in the skin of pseudo-albino compared to pigmented sole. The RNA-seq and qPCR results confirmed the importance of the α-MSH and ASIP system in pseudo-albinism although the specific involvement and relative importance of ASIP1 and ASIP2 remains to be investigated. In addition, transcripts encoding the enzymes GTP cyclohydrolase I (GCH1) and sepiapterin reductase (SPRB), involved in the synthesis of eumelanin in melanocytes and sepiapterin in xanthophores^37^, were strongly down-regulated in the pseudo-albino compared to the pigmented sole (Table 1, Fig. 5b). The apparent down-regulation of pigment biosynthesis fits well with the lack of visible xanthophores in pseudo-albino juvenile sole (Fig. 1) and is reminiscent of what has previously been reported for pseudo-albino larval sole^2^. Transcripts for enzymes modulating hypoxanthine biosynthesis (the main pigment in iridophores) such as *nt5c2*, *adad2*, *pnp* and *hprt1* were also different between pseudo-albino and pigmented control fish. Finally, the detected up-regulation of melanoregulin and down-regulation of stathmin-4^38,39^ suggests that disruption of pigment dispersion also occurs in pseudo-albino sole juveniles, in line with the observed round melanophores in the pseudo-albino juvenile (Fig. 1) and larval skin^2^. The results of the morphological and expression data suggest that there is a modified regulation of key steps for pigment biosynthesis and dispersion in the skin chromatophores of pseudo-albino sole.

RNA-seq suggested that retinoic acid (RA) metabolism was down-regulated in the skin of pseudo-albino juvenile sole compared to normally pigmented animals (Table 1). Vitamin A and the active form, RA, have previously been shown to be regulators of asymmetric pigmentation and photosensing in flatfish^9,40^, although vitamin A or all-trans-RA treatments did not induce malpigmentation in sole larvae^41^. A significant up-regulation of the RA-synthesizing enzyme *aldh1a3* was found in the pseudo-albino gut (Table 1, Fig. 5c), and this enzyme has previously been suggested to influence morphogenesis in developing sensory epithelia^42^. The potential link and functional significance of the up-regulated *aldh1a3* expression, RA-signalling and the structural changes detected in the gut of pseudo-albino sole remains to be investigated.

Expression data also suggested a dysregulation of a high number of transcription factors (TFs) in pseudo-albino sole, including members of the homeobox family (*hoxc13a*, *hoxC12a*, *hoxb1b*, *hoxb6b*, *hoxd4b*), the homeodomain-containing transcription factor *msx1*, the forkhead box protein *foxd5*, fibroblast growth factors (*fgf3*, *fgf4*, *fgf23*), sox proteins (*sox3*, *sox5*, *sox10*) and the transcript encoding LIM/homeobox protein (*lhx1*) (Table 1, Fig. 5). Some of these TFs are associated with skin development, skin regeneration and skin pigmentary anomalies in fish^43–45^ and may point to signalling pathways that are disrupted in the pseudo-albino in relation to pigmented sole, while possible associations with morphological or physiological differences remain to be investigated.

In this study, a significant increase in mucus-producing goblet cells was observed in the skin (but not in the gut) of pseudo-albino fish, along with changes in gene expression of several mucin forms in the skin or gut and significant up-regulation of *galnt17*, encoding an enzyme involved in mucin biosynthesis, in the gut (Table 1, Fig. 5). The mucus layer is known to contribute to the effectiveness of the gut and skin as physical and defensive barriers^46^. Previous correlations have been reported between mucins and the immune status of fish e.g.^47,48^, and the detected changes in gene expression for mucins and multiple components of the immune system (Table 1, Fig. 5) suggest that defensive systems differ between pseudo-albino and normally pigmented sole. In addition, the significant structural changes detected in pseudo-albino sole (e.g. a thicker gut muscle wall, Fig. 2) and global changes in expression of genes related to muscle contraction, ion transport or acid-base balance in the gut or skin structure and differentiation (Table 1, Fig. 4) also support the hypothesis that altered pigmentation in pseudo-albino sole might be the visible expression of a far more extensive syndrome affecting tissue morphology, physiology, innate immunity and potentially also their microbiome.

To test this hypothesis, whole bacterial microbiome characterization of the skin and gut of pseudo-albino fish compared to pigmented control fish was established for the first time. The results suggested that the skin and gut have different microbiomes and that the later also varies with pigmentation phenotypes. The sole skin 16 S rRNA microbiome was mainly composed of members of the *Proteobacteria* and *Firmicutes* phyla followed by smaller contributions from *Actinobacteria* and *Bacteroidetes*. This general distribution and the high diversity in the number of genera agrees with that reported for skin microbiomes of several other fish species^49–51^. The individual variability between replicate skin libraries is in line with previous observation of fish skin microbiomes at intra- and inter-species levels^49,51^, that were proposed to result from environmental or specific individual characteristics such as mucus composition or antimicrobial properties. The characterized juvenile sole skin microbiome was dominated by Gram negative bacteria (61% on average), as reported for Western mosquitofish^50^, which contrasted with the tendency in human and terrestrial animals for the skin microbiomes to be dominated by Gram positive bacteria.

The juvenile sole gut microbiome had a very low diversity and was mainly composed of bacteria from the genera *Mycoplasma* (*Tenericutes* phylum) and *Vibrio* (*Proteobacteria*) - Figs 6–7. These genera and the low diversity seems to be characteristic of fish gut microbiomes^20,52,53^. In the present study, culture-independent, high-throughput sequencing of the 16 S rRNA gene was used, as this is one of the most widely used markers in bacterial microbiome studies, mainly due to its universal taxonomic distribution and high database availability^52,54^. To minimize the potential limitations of this marker, due to its limited resolution of relative abundances in complex bacterial populations or to distinguish reads at a species level^52,54^, RT-PCR, cloning, Sanger sequencing and qPCR were also used. The relative abundance of the two main genera in the gut microbiomes of pseudo-albino or pigmented sole gut (which were of low complexity) were determined by qPCR, which confirmed the presence in normally pigmented juvenile sole of bacteria from the *Vibrio* genus, one of the most common genera found in the core microbiomes of aquaculture marine fish^20,52,53^. The species prevalence in the pigmented sole gut was *V*. *scophthalmi* followed by the related species *V*. *ichthyoenteri*^55^. The species *Vibrio harveyi* or *V*. *alginolyticus*, reported as pathogens in fish including the Senegalese sole^56^, were not amplified in the present study. *V*. *schophthalmi* and *V*. *ichthyoenteri* are reported to be abundant in healthy reared flatfish, although some strains have been reported to be opportunistic pathogens in other flatfish species^53,57–60^. In Senegalese sole under normal or intensive aquaculture it appears that *V*. *schophthalmi* and *V*. *ichthyoenteri* may be part of the normal healthy gut flora, and the present study supports this as apart from the pigmentation phenotype no signs of disease were found in any of the groups of sole sampled.

*Mycoplasma* are small bacteria devoid of a cell wall, inhabiting mucous surfaces of humans or fish and are generally considered non-cultivable^61,62^. Recent culture-independent studies indicate that *Mycoplasma* may also be components of the healthy gut microbiota of a variety of fish species, with particular abundance in the gut of omnivorous or carnivorous species as is the case of Senegalese sole^53,60,63,64^. Although some species (e.g. *M*. *mobile*) can be pathogenic, most species including *M*. *microti* and *M*. *muris* identified in Senegaese sole appeared to have no harmful effects on the host and the specific *Mycoplasma* may be very host- and tissue-specific due to habitat specialization^63,65^.

A previous study of the Senegalese sole gut microbiome also identified bacteria of the *Vibrio* genera and *V*. *ichthyoenteri* group as prevalent in fish fed with commercial diets, although *Mycoplasma* bacteria (the prevalent genera in the present study in both pigmented or pseudo-albino sole) were not detected^58^. Divergence in the main identified bacterial genera between the previous and present study may derive from the approach and sample type as the previous study analysed bacteria pre-selected by culture methods from adult sole whole gut^58^ while the present study used culture-independent 16 S rRNA gene sequencing for the juvenile anterior gut. In a recent 16 S rRNA-seq study *Mycoplasma* was also identified as one of the main genera detected in the Senegalese sole gut^60^, although in older sole from the same origin as those in the present study *Mycoplasma* were much less abundant^66^. Previous studies have indicated that age, diet, season and gut region can modify the fish gut microbiome and sample variation is a limiting factor for inter-study comparisons. In this context, recent recommendations have been made for standardization from sampling to microbiome data analysis, nonetheless next generation sequencing is contributing to increase understanding of fish gut microbial ecology^20,52,53,58^. In this context, the present study characterizes the microbiomes of the dorsal skin and anterior gut of pseudo-albino or pigmented juvenile sole, which were reared, sampled and analysed under the same conditions.

It is interesting that a modification in the anterior gut microbiome was identified in the pseudo-albino juvenile sole compared to normally pigmented controls, with significant decreases in the levels of both *Vibrio* and *Mycoplasma* bacteria (Fig. 7) and of the total bacterial load in the pseudo-albino gut in relation to the normally pigmented sole. The present study did not establish the factors causing the change in the microbiome but differences in tissue structure (eg. mucous cell number in the skin) and gene expression between pseudo-albino and pigmented sole were associated with tissue modifications (eg. muscle thickness) that presumably influenced the composition of the colonizing microbiota in the gut. Future studies will be required to investigate physical, chemical and functional differences in the pseudo-albino gut compared to the pigmented sole, since specialization of the gut microbiome is likely to be influenced by its internal characteristics and for instance the growth of both *Vibrio* and *Mycoplasma* is highly influenced by pH^53,58,60,67^. In addition, the effect of the modified microbiome on gut gene expression, structure, and function (as it influences physiological processes such as digestion or metabolism and prevents colonization by pathogenic bacteria^53,68^) will be an area for future study.

We cannot rule out that the differences in the pseudo-albino juvenile sole microbiome might also be contributing to the maintenance of the pseudo-albinism phenotype. However, this phenotype is characterized by permanent alterations in pigmentation and the major identified causal factors (e.g. dietary imbalances or continuous illumination) appear to be only effective during a sensitive “pigmentation window” at the beginning of metamorphosis, probably due to disrupted thyroid-mediated signalling^2,5–10^. If the microbiome influences the pseudo-albinism phenotype this would have to occur in early development stages when pigmentation is programmed. It will be interesting in the future to investigate differences in the microbiome of premetamorphic sole larvae with experimentally induced pseudo-albinism.

## Conclusions

Significant alterations occurred in the skin and gut of pseudo-albino juvenile sole compared to pigmented sole and the changes were not limited to disrupted pigmentation (round-shaped melanophores and lack of xanthophores). In pseudo-albino sole an increase in mucous cells in the skin, thicker muscle walls and *lamina propria* in the gut and altered transcript expression related to the retinoic acid pathway, photosensing, skin differentiation or regeneration and gut muscle development and contraction suggests much more profound functional changes may occur. Finally, the significantly altered microbiome in the anterior gut of pseudo-albino juvenile sole, with a significant decrease in bacteria from the *Vibrio* and *Mycoplasma* genera in relation to the pigmented fish, suggests the tissue modifications in the pseudo-albino may influence the microbiome. The present study uncovers for the first-time evidence that skin pigmentation disorders in sole are associated with modifications in other skin processes and probably also the function of other barriers such as the gut and their associated microbiomes.

## Supplementary information

Main supplementary material of the manuscript

Supplementary table S1

Supplementary table S4

## Data Availability

The datasets generated during the current study were deposited in at the European Nucleotide Archive (ENA) under project numbers PRJEB29448 (metagenomics) and PRJEB29449 (transcriptomics), joined under the umbrella project PRJEB29749.
