# Supplementary material for: Understanding pseudo-albinism in sole (Solea senegalensis): a transcriptomics and metagenomics approach
Source: Sci Rep. 2019 Sep 20;9:13604. doi: 10.1038/s41598-019-49501-6 (PMC6754371; doi:10.1038/s41598-019-49501-6)
Supplement: Supplementary file 1 — Main supplementary material of the manuscript [file 41598_2019_49501_MOESM1_ESM.pdf]

Supplementary information

Understanding pseudo-albinism in sole (*Solea senegalensis*): a transcriptomics and metagenomics approach

Patricia I.S. Pinto[1], Cláudia C. Guerreiro[1], Rita A. Costa[1], Juan F. Martinez-Blanch[2], Carlos Carballo[3], Francisco Codoñer[2], Manuel Manchado[3,*], Deborah M. Power[1,*]

[1] Centre of Marine Sciences (CCMAR), Universidade do Algarve, Campus de Gambelas, 8005-139 Faro, Portugal;
[2] LifeSequencing, Parc Cientific Universidad De Valencia, Edif. 2, C/ Catedrático Agustín Escardino Benlloch, 9, 46980 Paterna, Spain
[3] Instituto de Investigación y Formación Agraria y Pesquera (IFAPA) Centro El Toruño, Camino Tiro de Pichon s/n, 11500 Cadiz, Spain

Corresponding authors*: dpower@ualg.pt; manuel.manchado@juntadeandalucia.es

Number of pages: 19
Number of tables: 10
Number of figures: 2

Supplementary tables

Supplementary Table S1. **List of primers used in this study** (*available as a separate MSExcel file).*

A- Gene-specific primers used in transcriptome confirmation by qPCR; B- Genus-specific primers used in bacterial genus/species confirmation; C- Genus-specific primers used in qPCR for microbiome results confirmation.

Supplementary Table S2. Sequencing statistics from the RNAseq analysis.
Number of raw and filtered reads and reads mapped to the *Solea senegalensis* representative transcriptome (in millions of reads, M) for the four RNA-seq libraries sequenced from dorsal skin or anterior gut of pigmented (P) or pseudo-albino (A) juvenile sole (n=4 individuals pooled). The average length of the cleaned reads is shown in base pairs (bp) and the total amount of sequencing data produced is shown in Giga bases (Gb).

	**Raw reads (M)**	**Filtered reads (M)**	**Mapped reads (M)**	**Av. Length (bp)**	**Total data (Gb)**
Skin P	123.8	112.8	91.8	67.2	7.6
Skin A	133.7	121.6	99.7	67.2	8.2
Gut P	129.1	118.5	98.5	67.4	8.0
Gut A	139.8	124.2	103.7	67.4	8.4
Total	526.4	477.2	393.6		32.1
Average	131.6	119.3	98.4	67.3	8.1

Supplementary Table S3. Summary of differential expression results between pseudo-albino and pigmented juvenile sole (considered as the control), in the dorsal skin or anterior gut transcriptomes.

Candidate differentially expressed transcripts (DETs) were identified with the RobiNA software and the EdgeR package, using FDR threshold of 0.05 and mapping reads against the *Solea senegalensis* representative transcriptome. Up regulated indicates transcripts with higher expression in skin compared to gut or in pseudo-albino compared to pigmented sole.

	**Skin *vs* Gut**		**Pseudo-albino *vs* Pigmented**					
	**DETs**	**%**	**DETs in skin**	**%**	**DETs in gut**	**%**	**Shared DETs**	**%**
**Up regulated**	14,222	54.7	140	51.7	67	30.6	104	44.6
**Down regulated**	11,775	45.3	131	48.3	152	69.4	129	55.4
**Total**	25,997		271	100	219	100	233	100

Supplementary table S4. Expression data and annotation of the transcripts detected to be differentially expressed between pigmented and pseudo-albino juvenile sole *(available as a separate MSExcel file)*.

Differential expression (DE) is presented in Log2 fold change (FC) between pseudo-albino and control pigmented fish (LogFC AP) for transcripts found to have significant changes in expression (FDR<0.05) in the skin, in the gut or in common irrespectively of the tissue ("shared" statistics).

Supplementary Table S5. Gene ontology (GO) biological processes overexpressed among the list of all 573 transcripts differentially expressed between pigmented and pseudo-albino sole.

Significantly enriched biological processes (FDR<0.05) are grouped according to functionally related networks (GO group) identified by ClueGO analysis. Groups are named after its most significant term (lowest FDR), highlighted in bold, chosen for representation in Figure 4. Functionally related groups are sorted by highest enrichment score, calculated as - Log2 (group FDR).

GOID	GO Term	Term FDR	Group FDR	Enrichment Score	GO Groups	% Genes	Nr. Genes
GO:0003012	**muscle system process**	2.3E-04	2.2E-04	12.1	17	8.3	9
GO:0050879	multicellular organismal movement	2.9E-04	2.2E-04	12.1	17	19.2	5
GO:0050881	musculoskeletal movement	2.9E-04	2.2E-04	12.1	17	19.2	5
GO:0003009	skeletal muscle contraction	3.1E-04	2.2E-04	12.1	17	21.7	5
GO:0006941	striated muscle contraction	4.3E-04	2.2E-04	12.1	17	12.5	6
GO:0006936	muscle contraction	4.8E-04	2.2E-04	12.1	17	8.7	9
GO:0060048	cardiac muscle contraction	1.0E-02	2.2E-04	12.1	17	11.5	3
GO:0006937	regulation of muscle contraction	1.6E-02	2.2E-04	12.1	17	8.3	3
GO:0090257	regulation of muscle system process	1.7E-02	2.2E-04	12.1	17	7.9	3
GO:0070588	**calcium ion transmembrane transport**	2.5E-03	7.6E-04	10.4	18	5.8	8
GO:0072511	divalent inorganic cation transport	3.4E-03	7.6E-04	10.4	18	4.7	9
GO:0070838	divalent metal ion transport	3.4E-03	7.6E-04	10.4	18	4.7	9
GO:0006816	calcium ion transport	3.6E-03	7.6E-04	10.4	18	5.1	8
GO:0034765	regulation of ion transmembrane transport	6.7E-03	7.6E-04	10.4	18	4.1	9
GO:0034762	regulation of transmembrane transport	6.7E-03	7.6E-04	10.4	18	4.0	9
GO:0072507	divalent inorganic cation homeostasis	9.5E-03	7.6E-04	10.4	18	4.5	7
GO:0055074	calcium ion homeostasis	1.5E-02	7.6E-04	10.4	18	4.4	6
GO:0072503	cellular divalent inorganic cation homeostasis	1.6E-02	7.6E-04	10.4	18	4.2	6
GO:0001508	**action potential**	2.9E-03	4.9E-03	7.7	16	13.3	4
GO:0070588	calcium ion transmembrane transport	2.5E-03	4.9E-03	7.7	16	5.8	8
GO:0006816	calcium ion transport	3.6E-03	4.9E-03	7.7	16	5.1	8
GO:0034765	regulation of ion transmembrane transport	6.7E-03	4.9E-03	7.7	16	4.1	9
GO:0034762	regulation of transmembrane transport	6.7E-03	4.9E-03	7.7	16	4.0	9
GO:0051899	membrane depolarization	7.5E-03	4.9E-03	7.7	16	13.6	3
GO:0086010	membrane depolarization during action potential	7.5E-03	4.9E-03	7.7	16	13.6	3
GO:0042391	regulation of membrane potential	9.2E-03	4.9E-03	7.7	16	4.5	7
GO:0042559	**pteridine-containing compound biosynthetic process**	2.8E-03	9.0E-03	6.8	10	27.3	3
GO:0042558	pteridine-containing compound metabolic process	6.4E-03	9.0E-03	6.8	10	15.0	3
GO:0006814	**sodium ion transport**	9.4E-03	1.0E-02	6.6	9	5.2	6
GO:0035725	sodium ion transmembrane transport	1.5E-02	1.0E-02	6.6	9	6.3	4
GO:0003006	**developmental process involved in reproduction**	1.0E-02	1.1E-02	6.5	5	6.0	5

GO:0071600	**otic vesicle morphogenesis**	1.0E-03	1.2E-02	6.4	19	13.9	5
GO:0043049	otic placode formation	2.2E-03	1.2E-02	6.4	19	16.7	4
GO:0030916	otic vesicle formation	2.4E-03	1.2E-02	6.4	19	14.3	4
GO:0042471	ear morphogenesis	2.7E-03	1.2E-02	6.4	19	8.0	6
GO:0042472	inner ear morphogenesis	2.7E-03	1.2E-02	6.4	19	8.1	6
GO:0071599	otic vesicle development	2.8E-03	1.2E-02	6.4	19	9.4	5
GO:0060788	ectodermal placode formation	3.3E-03	1.2E-02	6.4	19	12.5	4
GO:0009880	embryonic pattern specification	4.1E-03	1.2E-02	6.4	19	8.2	5
GO:0030878	thyroid gland development	4.5E-03	1.2E-02	6.4	19	17.6	3
GO:0071697	ectodermal placode morphogenesis	4.7E-03	1.2E-02	6.4	19	10.5	4
GO:0071696	ectodermal placode development	7.4E-03	1.2E-02	6.4	19	8.9	4
GO:0048839	inner ear development	9.9E-03	1.2E-02	6.4	19	4.4	7
GO:0043583	ear development	1.0E-02	1.2E-02	6.4	19	4.3	7
GO:0039021	pronephric glomerulus development	1.0E-02	1.2E-02	6.4	19	11.5	3
GO:0072175	epithelial tube formation	1.4E-02	1.2E-02	6.4	19	6.7	4
GO:0035148	tube formation	1.5E-02	1.2E-02	6.4	19	6.3	4
GO:0030901	midbrain development	1.6E-02	1.2E-02	6.4	19	8.6	3
GO:0007422	peripheral nervous system development	1.6E-02	1.2E-02	6.4	19	6.0	4
GO:0039019	pronephric nephron development	1.7E-02	1.2E-02	6.4	19	8.1	3
GO:0072006	nephron development	1.8E-02	1.2E-02	6.4	19	5.6	4
GO:0022037	metencephalon development	3.0E-02	1.2E-02	6.4	19	6.1	3
GO:0032835	glomerulus development	3.5E-02	1.2E-02	6.4	19	5.8	3
GO:0015696	**ammonium transport**	1.5E-02	1.4E-02	6.2	6	9.1	3
GO:0007601	**visual perception**	1.5E-02	1.4E-02	6.1	7	5.1	5
GO:0050953	sensory perception of light stimulus	1.5E-02	1.4E-02	6.1	7	5.0	5
GO:0014706	**striated muscle tissue development**	1.3E-02	1.5E-02	6.0	12	4.1	7
GO:0060537	muscle tissue development	1.3E-02	1.5E-02	6.0	12	4.1	7
GO:0007519	skeletal muscle tissue development	1.5E-02	1.5E-02	6.0	12	5.2	5
GO:0060538	skeletal muscle organ development	1.7E-02	1.5E-02	6.0	12	4.6	5
GO:0071774	**response to fibroblast growth factor**	1.4E-02	1.5E-02	6.0	11	6.8	4
GO:0044344	cellular response to fibroblast growth factor stimulus	1.4E-02	1.5E-02	6.0	11	6.8	4
GO:0008543	fibroblast growth factor receptor signaling pathway	1.4E-02	1.5E-02	6.0	11	6.8	4
GO:0001704	**formation of primary germ layer**	1.8E-02	1.5E-02	6.0	14	5.5	4
GO:0060216	definitive hemopoiesis	2.9E-02	1.5E-02	6.0	14	6.3	3
GO:0060215	primitive hemopoiesis	3.7E-02	1.5E-02	6.0	14	5.6	3
GO:0007492	endoderm development	4.9E-02	1.5E-02	6.0	14	4.8	3
GO:0072522	**purine-containing compound biosynthetic process**	1.6E-02	1.6E-02	6.0	15	4.1	6
GO:0046068	cGMP metabolic process	1.7E-02	1.6E-02	6.0	15	8.1	3
GO:0006182	cGMP biosynthetic process	1.7E-02	1.6E-02	6.0	15	8.1	3

GO:0052652	cyclic purine nucleotide metabolic process	4.8E-02	1.6E-02	6.0	15	4.8	3
GO:0009190	cyclic nucleotide biosynthetic process	4.9E-02	1.6E-02	6.0	15	4.8	3
GO:0090092	**regulation of transmembrane receptor protein serine/threonine kinase signaling pathway**	2.5E-02	2.6E-02	5.3	2	4.1	5
GO:0030593	**neutrophil chemotaxis**	2.8E-02	4.0E-02	4.6	13	6.4	3
GO:0071621	granulocyte chemotaxis	3.0E-02	4.0E-02	4.6	13	6.1	3
GO:1990266	neutrophil migration	3.6E-02	4.0E-02	4.6	13	5.7	3
GO:0097530	granulocyte migration	3.8E-02	4.0E-02	4.6	13	5.5	3
GO:0050851	**antigen receptor-mediated signaling pathway**	4.2E-02	4.1E-02	4.6	1	5.2	3
GO:0006006	**glucose metabolic process**	1.8E-02	4.1E-02	4.6	8	7.7	3
GO:0019318	hexose metabolic process	4.6E-02	4.1E-02	4.6	8	4.9	3
GO:0010038	**response to metal ion**	4.1E-02	4.1E-02	4.6	4	5.3	3
GO:1902476	**chloride transmembrane transport**	4.5E-02	4.2E-02	4.6	3	5.0	3

Supplementary Table S6. Significantly enriched biological processes (FDR<0.05), global or tissue-specific, grouped according to functionally related networks (GO group) identified by ClueGO analysis.
Significantly enriched biological processes (FDR<0.05) are grouped according to functionally related networks (GO group) identified by ClueGO analysis. Groups are named after its most significant term (lowest FDR), highlighted in bold, chosen for representation in Figure 4. Functionally related groups are sorted by highest enrichment score, calculated as - Log2 (group FDR).

A. Gene ontology (GO) biological processes overexpressed in list SKIN SPECIFIC DET (differentially expression between pseudo-albino and pigmented sole, detected specifically in the skin):

GOID	GOTerm	Term FDR	Group FDR	Enrichment Score	GO Groups	% Genes	Nr. Genes
GO:0042559	**pteridine-containing compound biosynthetic process**	43.7E-6	195.4E-6	12.3	2	27.3	3
GO:0042558	pteridine-containing compound metabolic process	146.6E-6	195.4E-6	12.3	2	15.0	3
GO:0009880	**embryonic pattern specification**	2.7E-3	2.7E-3	8.5	1	4.9	3

B. Gene ontology (GO) biological processes overexpressed in list GUT SPECIFIC DET (differentially expression between pseudo-albino and pigmented sole, detected specifically in the gut):

GOID	GOTerm	Term FDR	Group FDR	Enrichment Score	GO Groups	% Genes	Nr. Genes
GO:0006936	**muscle contraction**	6.9E-6	14.1E-6	16.1	3	5.8	6
GO:0003012	muscle system process	7.2E-6	14.1E-6	16.1	3	5.5	6
GO:0003009	skeletal muscle contraction	7.8E-6	14.1E-6	16.1	3	17.4	4
GO:0050879	multicellular organismal movement	8.7E-6	14.1E-6	16.1	3	15.4	4
GO:0050881	musculoskeletal movement	8.7E-6	14.1E-6	16.1	3	15.4	4
GO:0006941	striated muscle contraction	10.2E-6	14.1E-6	16.1	3	10.4	5
GO:0007519	**skeletal muscle tissue development**	821.1E-6	135.7E-6	12.8	2	4.1	4
GO:0048747	muscle fiber development	2.8E-3	135.7E-6	12.8	2	4.5	3
GO:0042471	**ear morphogenesis**	3.1E-3	3.1E-3	8.3	1	4.0	3
GO:0042472	inner ear morphogenesis	3.4E-3	3.1E-3	8.3	1	4.1	3

C. Gene ontology (GO) biological processes overexpressed in list SHARED DET (differentially expression between pseudo-albino and pigmented sole, detected in both skin and gut):

GOID	GOTerm	Term FDR	Group FDR	Enrichment Score	GO Groups	% Genes	Nr. Genes
GO:0050953	**sensory perception of light stimulus**	1.1E-04	1.5E-04	12.7	2	5.0	5
GO:0007601	visual perception	1.9E-04	1.5E-04	12.7	2	5.1	5
GO:0003006	**developmental process involved in reproduction**	4.7E-04	4.7E-04	11.1	1	4.8	4

Supplementary Table S7. Sequencing statistics from 16S rRNA gene microbiome analyses
Number of raw, paired end and filtered reads are shown for each microbiome library from the skin or gut DNA of pigmented (P) or pseudo-albino (A) juvenile sole (2 microbiome libraries/group, pooling 2 individuals each) or from their food and environmental water (H2O). Also shown are the average length of the cleaned reads (in base pairs, bp), total amount of sequencing data produced (in mega bases, Mb) and their average quality (Phred / Q value).

	Raw reads	Paired-end reads	Filtered reads	Av. Length (bp)	Total Mb	Av. Qual.
Gut P1	148,342	71,946	67,513	436.5	29.5	37.3
Gut P2	84,014	37,550	34,540	432.2	14.9	37.3
Gut A1	125,724	54,931	50,245	427.6	21.5	37.2
Gut A2	129,374	61,553	57,283	435.5	25.0	37.2
Skin P1	163,472	54,292	37,936	379.9	14.4	37.1
Skin P2	211,364	63,523	56,292	415.6	23.4	37.0
Skin A1	264,806	90,215	69,003	390.7	27.0	37.2
Skin A2	153,088	55,727	35,663	392.8	14.0	37.2
Water	210,714	101,154	92,067	426.2	39.2	37.1
Food	175,440	86,439	75,896	419.2	31.8	37.2
Total	1,666,338	677,330	576,438		240.7	

Supplementary Table S8. Main bacterial genera detected by 16s rRNA gene sequencing.
Proportions of identified genera in each library are presented in percentage. Only genera detected at a minimum of 1% in the sum of all libraries are presented but Shannon diversity indexes were calculated based on all detected genera. < 1% relative proportions in each library are shaded in grey. The % of the most abundant genus detected in each library is highlighted in bold.

**OTU identification**	**Microbiome libraries**									
**Main genera**	**Water**	**Food**	**Sk P1**	**Sk P2**	**Sk A1**	**Sk A2**	**Gut P1**	**Gut P2**	**Gut A1**	**Gut A2**
Endozoicomonas	**12.5**	0.0	0.1	0.0	0.2	0.0	0.0	0.0	0.0	0.0
Spongiibacter	7.4	0.0	2.7	0.7	1.9	1.0	0.0	0.0	0.0	0.0
Thioprofundum	4.3	0.0	0.5	0.7	0.1	0.3	0.0	0.0	0.0	0.0
Candidatus Limnoluna	3.5	0.0	0.0	0.0	0.0	0.0	0.0	0.0	0.0	0.0
Gimesia	2.5	0.0	0.2	0.0	0.0	0.0	0.0	0.0	0.0	0.0
Candidatus Pelagibacter	2.2	0.0	0.0	0.0	0.0	0.0	0.0	0.0	0.0	0.0
Rubinisphaera	2.1	0.0	0.0	0.1	0.0	0.0	0.0	0.0	0.0	0.0
Rhodoluna	2.1	0.0	0.1	0.0	0.0	0.1	0.0	0.0	0.0	0.0
Mesoflavibacter	1.4	0.0	0.5	0.7	0.3	0.0	0.0	0.0	0.0	0.0
Pontimonas	1.3	0.0	0.0	0.0	0.0	0.0	0.0	0.0	0.0	0.0
Kangiella	1.0	0.0	0.0	0.8	0.4	0.1	0.0	0.0	0.0	0.0
Herbiconiux	1.0	0.0	0.0	0.0	0.0	0.0	0.0	0.0	0.0	0.0
Pseudoalteromonas	0.3	0.0	1.3	0.3	0.4	2.4	0.0	0.0	0.0	0.0
Geobacter	0.2	0.0	0.0	1.4	0.2	0.1	0.0	0.0	0.0	0.0
Rhizobium	0.2	0.0	1.0	0.0	0.0	0.0	0.0	0.0	0.0	0.0
Oceanospirillum	0.1	0.0	1.6	0.1	0.3	0.1	0.0	0.0	0.0	0.0
Acinetobacter	0.1	5.8	3.1	0.6	0.8	1.5	0.0	0.0	0.1	0.0
Trichocoleus	0.1	1.1	0.0	0.0	0.0	0.0	0.0	0.0	0.0	0.0
Staphylococcus	0.1	0.2	6.3	1.0	10.2	1.7	0.0	0.0	0.1	0.0
Clostridium	0.0	0.2	0.4	0.0	0.0	1.6	0.0	0.0	0.0	0.0
Leucothrix	0.0	0.0	0.0	0.4	3.1	0.0	0.0	0.0	0.0	0.0
Aerosakkonema	0.0	4.3	0.1	0.0	2.1	0.3	0.0	0.0	0.0	0.0
Litoreibacter	0.0	0.0	0.0	0.0	1.0	0.0	0.0	0.0	0.0	0.0
Faecalibacterium	0.0	0.0	0.0	0.0	0.2	8.0	0.0	0.0	0.0	0.0
Geojedonia	0.0	0.0	0.0	0.5	0.1	3.0	0.0	0.0	0.0	0.0
Weissella	0.0	7.4	0.0	0.0	0.0	0.0	0.0	0.0	0.0	0.0

Photobacterium	0.0	3.4	0.0	0.0	0.0	0.0	0.0	0.0	0.0	0.0
Prevotella	0.0	0.1	3.8	0.1	0.8	0.6	0.0	0.0	0.0	0.0
Arthrospira	0.0	**40.0**	0.0	0.0	0.0	0.0	0.0	0.0	0.0	0.0
Facklamia	0.0	1.1	0.0	0.0	0.0	0.0	0.0	0.0	0.0	0.0
Pediococcus	0.0	3.4	0.0	0.0	0.0	0.0	0.0	0.0	0.0	0.0
Rivularia	0.0	2.1	0.0	0.0	0.0	0.0	0.0	0.0	0.0	0.0
Geobacillus	0.0	1.0	0.0	0.0	0.0	0.0	0.0	0.0	0.0	0.0
Lautropia	0.0	0.0	1.1	0.0	0.0	0.0	0.0	0.0	0.0	0.0
Roseburia	0.0	0.0	0.0	0.0	0.0	2.5	0.0	0.0	0.0	0.0
Collinsella	0.0	0.0	0.3	0.0	0.0	1.6	0.0	0.0	0.0	0.0
Neisseria	0.0	0.0	1.7	0.0	0.0	0.3	0.0	0.0	0.0	0.0
Marinobacter	4.6	0.0	1.5	0.6	1.6	0.7	0.0	0.0	0.0	0.0
Leisingera	2.2	0.0	0.0	0.1	0.0	0.0	0.0	0.0	0.0	0.0
Paracoccus	0.6	0.0	1.7	0.0	0.1	0.0	0.0	0.0	0.0	0.0
Rubritalea	0.5	0.0	2.3	3.0	6.3	1.6	0.0	0.0	0.0	0.0
Sulfitobacter	0.4	0.0	0.3	2.6	0.5	0.9	0.0	0.0	0.0	0.0
Alteromonas	0.2	0.0	5.9	0.5	1.8	0.3	0.0	0.0	0.0	0.0
Streptococcus	0.1	1.9	5.8	0.2	1.4	1.1	0.0	0.0	0.1	0.0
Moraxella	0.1	0.0	4.5	1.2	6.9	1.4	0.0	0.0	0.0	0.0
Reichenbachiella	0.1	0.0	0.0	3.7	0.1	0.6	0.0	0.0	0.0	0.0
Corynebacterium	0.1	0.1	1.6	0.1	1.1	0.5	0.0	0.0	0.0	0.0
Lactobacillus	0.0	5.7	0.0	0.0	0.1	1.8	0.0	0.0	0.0	0.0
Dokdonia	1.4	0.0	0.4	1.1	0.6	0.4	0.0	0.0	0.0	0.0
Lewinella	1.2	0.0	0.7	2.5	0.6	2.0	0.0	0.0	0.0	0.0
Aureispira	1.0	0.0	0.6	18.9	4.5	1.4	0.0	0.1	0.0	0.0
Flexibacter	0.5	0.0	0.0	3.0	0.3	0.0	0.0	0.0	0.0	0.0
Gaetbulibacter	0.2	0.0	0.0	1.1	0.1	0.2	0.0	0.0	0.0	0.0
Aliiroseovarius	0.1	0.0	0.7	4.3	16.5	0.4	0.0	0.0	0.0	0.0
Veillonella	0.0	0.0	4.1	0.1	2.0	5.1	0.0	0.0	0.0	0.0
Enterococcus	0.0	4.9	0.0	0.0	0.0	0.0	0.0	0.0	0.0	0.0
Eubacterium	0.0	0.0	0.0	0.0	0.0	8.1	0.0	0.0	0.0	0.0
Winogradskyella	0.6	0.1	0.0	0.9	5.4	0.5	0.0	0.0	0.0	0.0
Propionibacterium	0.1	0.0	1.3	0.3	1.3	0.5	0.0	0.0	0.1	0.0
Citrobacter	0.0	0.0	0.6	0.8	0.2	0.2	0.0	0.0	10.8	0.0

Alcanivorax	4.2	0.0	1.0	0.7	1.5	0.0	0.0	0.1	0.0	0.0
Pseudomonas	0.4	0.2	1.7	0.5	0.9	0.4	0.0	0.0	0.1	0.0
Blautia	0.0	0.0	0.0	0.0	0.1	1.2	0.0	0.0	0.0	0.0
Bacillus	0.1	0.6	**16.0**	2.0	2.2	2.6	0.0	0.0	0.2	0.1
Bacteroides	0.0	0.1	1.3	0.1	0.4	9.6	0.0	0.0	0.0	0.0
Escherichia	0.0	0.0	0.4	0.5	0.4	5.9	0.0	0.0	0.0	0.0
Lacinutrix	1.0	0.0	0.0	**25.4**	2.9	0.6	0.0	0.1	0.0	0.0
Bifidobacterium	0.0	0.1	1.2	0.2	3.1	8.6	0.0	0.1	0.2	0.0
Brevinema	0.0	0.0	0.6	0.2	1.4	0.0	0.4	19.6	0.0	0.0
Vibrio	1.0	0.1	3.4	1.0	1.1	0.4	27.5	23.5	0.2	0.2
Mycoplasma	0.0	0.1	3.5	0.1	1.4	1.8	**71.4**	**55.8**	**86.6**	**99.5**

Supplementary Table S9. Main bacterial species detected by 16s rRNA gene sequencing.
Proportions of identified species in each library are presented in percentage. Only species detected at a minimum of 1% in the sum of all libraries are presented but Shannon diversity indexes were calculated based on all detected species. < 1% relative proportions in each library are shaded in grey. The % of the most abundant species detected in each library is highlighted in bold.

**OTU identification**	**Microbiome libraries**									
**Main species**	**Water**	**Food**	**Sk P1**	**Sk P2**	**Sk A1**	**Sk A2**	**Gut P1**	**Gut P2**	**Gut A1**	**Gut A2**
Endozoicomonas euniceicola	**12.5**	0.0	0.1	0.0	0.2	0.0	0.0	0.0	0.0	0.0
Spongiibacter marinus	7.2	0.0	2.7	0.7	1.9	1.0	0.0	0.0	0.0	0.0
Marinobacter goseongensis	4.1	0.0	1.4	0.4	1.6	0.0	0.0	0.0	0.0	0.0
Alcanivorax borkumensis	3.9	0.0	1.0	0.6	1.4	0.0	0.0	0.0	0.0	0.0
Candidatus Limnoluna rubra	3.5	0.0	0.0	0.0	0.0	0.0	0.0	0.0	0.0	0.0
Thioprofundum lithotrophicum	3.2	0.0	0.5	0.4	0.1	0.3	0.0	0.0	0.0	0.0
Gimesia maris	2.5	0.0	0.2	0.0	0.0	0.0	0.0	0.0	0.0	0.0
Rubinisphaera brasiliensis	2.1	0.0	0.0	0.1	0.0	0.0	0.0	0.0	0.0	0.0
Candidatus Rhodoluna limnophila	2.1	0.0	0.1	0.0	0.0	0.1	0.0	0.0	0.0	0.0
Leisingera caerulea	2.1	0.0	0.0	0.0	0.0	0.0	0.0	0.0	0.0	0.0
Dokdonia donghaensis	1.4	0.0	0.4	1.0	0.6	0.4	0.0	0.0	0.0	0.0
Mesoflavibacter zeaxanthinifaciens	1.4	0.0	0.5	0.7	0.3	0.0	0.0	0.0	0.0	0.0
Candidatus Pelagibacter ubique	1.4	0.0	0.0	0.0	0.0	0.0	0.0	0.0	0.0	0.0
Pontimonas salivibrio	1.3	0.0	0.0	0.0	0.0	0.0	0.0	0.0	0.0	0.0
Thioprofundum hispidum	1.1	0.0	0.0	0.3	0.0	0.0	0.0	0.0	0.0	0.0
Aureispira maritima	1.0	0.0	0.6	18.9	4.5	1.4	0.0	0.1	0.0	0.0
Lacinutrix sp. 5H-3-7-4	1.0	0.0	0.0	**24.8**	2.8	0.6	0.0	0.1	0.0	0.0
Lewinella nigricans	0.9	0.0	0.5	1.4	0.4	2.0	0.0	0.0	0.0	0.0
Flexibacter litoralis	0.5	0.0	0.0	2.8	0.3	0.0	0.0	0.0	0.0	0.0
Winogradskyella jejuensis	0.4	0.1	0.0	0.0	5.3	0.3	0.0	0.0	0.0	0.0
Sulfitobacter pontiacus	0.2	0.0	0.3	1.0	0.5	0.5	0.0	0.0	0.0	0.0
Streptococcus dentisani	0.1	0.0	0.2	0.1	1.3	0.8	0.0	0.0	0.1	0.0
Acinetobacter junii	0.1	0.0	2.5	0.6	0.6	0.1	0.0	0.0	0.0	0.0
Reichenbachiella faecimaris	0.1	0.0	0.0	3.7	0.1	0.6	0.0	0.0	0.0	0.0
Trichocoleus desertorum	0.1	1.1	0.0	0.0	0.0	0.0	0.0	0.0	0.0	0.0
Aliiroseovarius sediminilitoris	0.1	0.0	0.7	0.2	16.5	0.4	0.0	0.0	0.0	0.0

Oceanospirillum beijerinckii	0.1	0.0	1.6	0.1	0.3	0.1	0.0	0.0	0.0	0.0
Propionibacterium acnes	0.1	0.0	1.2	0.3	1.3	0.5	0.0	0.0	0.1	0.0
Staphylococcus epidermidis	0.1	0.0	6.0	0.0	9.8	1.7	0.0	0.0	0.1	0.0
Moraxella porci	0.0	0.0	4.5	1.2	6.7	1.2	0.0	0.0	0.0	0.0
Rubritalea marina	0.0	0.0	1.3	2.6	5.3	1.4	0.0	0.0	0.0	0.0
Alteromonas tagae	0.0	0.0	5.9	0.5	1.8	0.3	0.0	0.0	0.0	0.0
Leucothrix mucor	0.0	0.0	0.0	0.4	3.1	0.0	0.0	0.0	0.0	0.0
Pseudomonas punonensis	0.0	0.0	1.3	0.4	0.4	0.3	0.0	0.0	0.0	0.0
Geobacter grbiciae	0.0	0.0	0.0	1.4	0.2	0.0	0.0	0.0	0.0	0.0
Bifidobacterium longum	0.0	0.0	0.8	0.1	0.4	4.6	0.0	0.0	0.1	0.0
Aerosakkonema funiforme	0.0	4.3	0.1	0.0	2.1	0.3	0.0	0.0	0.0	0.0
Pseudoalteromonas arabiensis	0.0	0.0	0.0	0.0	0.0	1.5	0.0	0.0	0.0	0.0
Mycoplasma muris	0.0	0.0	3.2	0.1	1.3	1.6	**68.8**	**52.8**	**83.6**	**96.2**
Litoreibacter halocynthiae	0.0	0.0	0.0	0.0	1.0	0.0	0.0	0.0	0.0	0.0
Faecalibacterium prausnitzii	0.0	0.0	0.0	0.0	0.2	8.0	0.0	0.0	0.0	0.0
Veillonella dispar	0.0	0.0	4.1	0.0	1.8	4.8	0.0	0.0	0.0	0.0
Brevinema andersonii	0.0	0.0	0.6	0.2	1.4	0.0	0.4	19.6	0.0	0.0
Pseudoalteromonas marina	0.0	0.0	1.1	0.3	0.3	0.2	0.0	0.0	0.0	0.0
Vibrio parahaemolyticus	0.0	0.0	1.0	0.0	0.0	0.0	26.4	0.3	0.2	0.0
Aliiroseovarius crassostreae	0.0	0.0	0.0	4.1	0.0	0.0	0.0	0.0	0.0	0.0
Vibrio scophthalmi	0.0	0.0	0.0	0.0	0.2	0.0	0.1	1.8	0.0	0.0
Geojedonia litorea	0.0	0.0	0.0	0.5	0.1	3.0	0.0	0.0	0.0	0.0
Escherichia fergusonii	0.0	0.0	0.4	0.5	0.4	5.9	0.0	0.0	0.0	0.0
Facklamia tabacinasalis	0.0	1.1	0.0	0.0	0.0	0.0	0.0	0.0	0.0	0.0
Arthrospira platensis	0.0	**40.0**	0.0	0.0	0.0	0.0	0.0	0.0	0.0	0.0
Acinetobacter johnsonii	0.0	0.3	0.0	0.0	0.0	1.4	0.0	0.0	0.0	0.0
Enterococcus saccharolyticus	0.0	4.7	0.0	0.0	0.0	0.0	0.0	0.0	0.0	0.0
Vibrio atypicus	0.0	0.0	0.7	0.2	0.0	0.0	0.0	21.0	0.0	0.1
Mycoplasma microti	0.0	0.0	0.3	0.0	0.1	0.1	2.6	3.0	2.9	3.3
Weissella confusa	0.0	7.1	0.0	0.0	0.0	0.0	0.0	0.0	0.0	0.0
Citrobacter freundii	0.0	0.0	0.6	0.8	0.1	0.2	0.0	0.0	10.8	0.0
Lactobacillus rogosae	0.0	0.0	0.0	0.0	0.0	1.6	0.0	0.0	0.0	0.0
Lactobacillus amylovorus	0.0	1.8	0.0	0.0	0.0	0.0	0.0	0.0	0.0	0.0
Bacteroides dorei	0.0	0.0	0.0	0.0	0.3	8.6	0.0	0.0	0.0	0.0

Bacillus toyonensis	0.0	0.0	**15.6**	2.0	1.9	2.6	0.0	0.0	0.2	0.1
Acinetobacter schindleri	0.0	4.8	0.0	0.0	0.0	0.0	0.0	0.0	0.0	0.0
Streptococcus cristatus	0.0	0.0	5.1	0.0	0.0	0.0	0.0	0.0	0.0	0.0
Pediococcus pentosaceus	0.0	3.3	0.0	0.0	0.0	0.0	0.0	0.0	0.0	0.0
Photobacterium iliopiscarium	0.0	2.7	0.0	0.0	0.0	0.0	0.0	0.0	0.0	0.0
Rivularia sp. PCC 7116	0.0	2.1	0.0	0.0	0.0	0.0	0.0	0.0	0.0	0.0
Streptococcus porcorum	0.0	1.6	0.0	0.0	0.0	0.0	0.0	0.0	0.0	0.0
Bifidobacterium adolescentis	0.0	0.0	0.2	0.1	0.1	3.2	0.0	0.0	0.0	0.0
Lautropia mirabilis	0.0	0.0	1.1	0.0	0.0	0.0	0.0	0.0	0.0	0.0
Eubacterium eligens	0.0	0.0	0.0	0.0	0.0	6.5	0.0	0.0	0.0	0.0
Collinsella aerofaciens	0.0	0.0	0.3	0.0	0.0	1.6	0.0	0.0	0.0	0.0
Roseburia intestinalis	0.0	0.0	0.0	0.0	0.0	1.2	0.0	0.0	0.0	0.0
Vibrio gigantis	0.0	0.0	1.5	0.4	0.0	0.3	0.0	0.0	0.0	0.0
Prevotella melaninogenica	0.0	0.0	2.7	0.0	0.2	0.2	0.0	0.0	0.0	0.0
Bifidobacterium animalis	0.0	0.0	0.0	0.0	2.4	0.0	0.0	0.0	0.0	0.0
Paracoccus aminovorans	0.0	0.0	1.7	0.0	0.1	0.0	0.0	0.0	0.0	0.0
Corynebacterium accolens	0.0	0.0	1.1	0.0	0.0	0.0	0.0	0.0	0.0	0.0
shannon only genera 1%	2.9	0.0	0.5	1.2	0.8	0.4	0.0	0.0	0.0	0.0
**Shannon diversity index (SHI)**	4.80	3.24	3.50	3.99	4.15	0.67	1.31	3.82	0.19	0.84
**Average Shannon diversity index**	4.80	3.24	3.74		2.41		2.56		0.52	

Supplementary Table S10. Accession numbers of the agouti-signaling proteins (ASIP1/ASIP2) and Agouti-related proteins (AGRP/AGRP2) used in the phylogenetic analysis.
Accession numbers of duplicate genes in this table are presented in the same order as in the phylogenetic tree (Supplementary Figure S2). n.i.: not identified.

	ASIP/ASIP1	AGRP	ASIP2/AGRP2
Homo sapiens	ENSP00000364092	ENSP00000290953	n.i.
Mus musculus	ENSMUSP00000029123	ENSMUSP00000142044	n.i.
Rattus norvegicus	ENSRNOP00000023905	ENSRNOP00000056384	n.i.
Sus scrofa	ENSSSCP00000007749	ENSSSCP00000002994	n.i.
Bos taurus	NP_996674	ENSBTAP00000019347	n.i.
Vulpes vulpes	CAA71004	XP_025867287	n.i.
Gallus gallus	ENSGALP00000056224	ENSGALP00000003505	n.i.
Xenopus tropicalis	FAA00757	XP_017948975	n.i.
Latimeria chalumnea	XP_005986820	JH128494	n.i.
Tetraodon nigroviridis	H3C4B2	ENSTNIP00000013124	ENSTNIP00000010647, FAA00756
Takifugu rubripes	ENSTRUP00000008132	NP_001092125	NP_001092124, NP_001092126
Gasterosteus aculeatus	CCB84813	FAA00758	FAA00755, FAA00754
Dicentrarchus labrax	CCW43207	CCF78543	CCW03299, CCF78544
Lateolabrax japonicus	n.i.	AIJ03132	AIJ03133
Sparus aurata	n.i.	AMZ00814	AWX49633
Larimichthys crocea	XP_010749988	n.i.	XP_010741851, XP_019114556
Oreochromis niloticus	XP_003448419	XP_019216758	FAA00764
Haplochromis burtoni	NP_001273227,	XP_005929271	XP_005918457, XP_005939107
Onchorynchus mykiss	NP_001171707, XP_021464730	XP_021450164	XP_021445599
Salmo salar	n.i.	NP_001140149	NP_001140150
Scophthalmus maximus	CCD42017	AWP01088	AWP04382
Platichthys stellatus	n.i.	APY24031	APY24032
Solea senegalensis	CCD42018	n.i.	solea_v4.1_unigene59061
Gadus morhua	FAA00761	FAA00762	FAA00759
Danio rerio	ENSDARP00000100167	NP_001314941	ENSDARP00000151238
Carassius auratus	CAH60802	CAD88211	XP_026051772
Scleropages formosus	XP_018597801	KPP66067	KPP62628
Astyanax mexicanus	XP_007258567	XP_0225227877	ENSAMXP00000019735, XP_022533387
Lepisosteus oculatus	XP_015220500	XP_006641582	XP_015209867
Callorhinchus milii	XP_007907480	XP_007887790	n.i.

Supplementary Figures

Supplementary Figure S1. Rarefaction curves for the ten microbiome libraries.
A total of 4 pigmented (P) sole or 4 pseudo-albino (A) sole were used to generate 2 libraries per analyzed group, as well as their food and tank water. The number of observed OTUs (operational taxonomic units) at the genera level is plotted against the number of 16S rRNA gene sequences obtained per library.

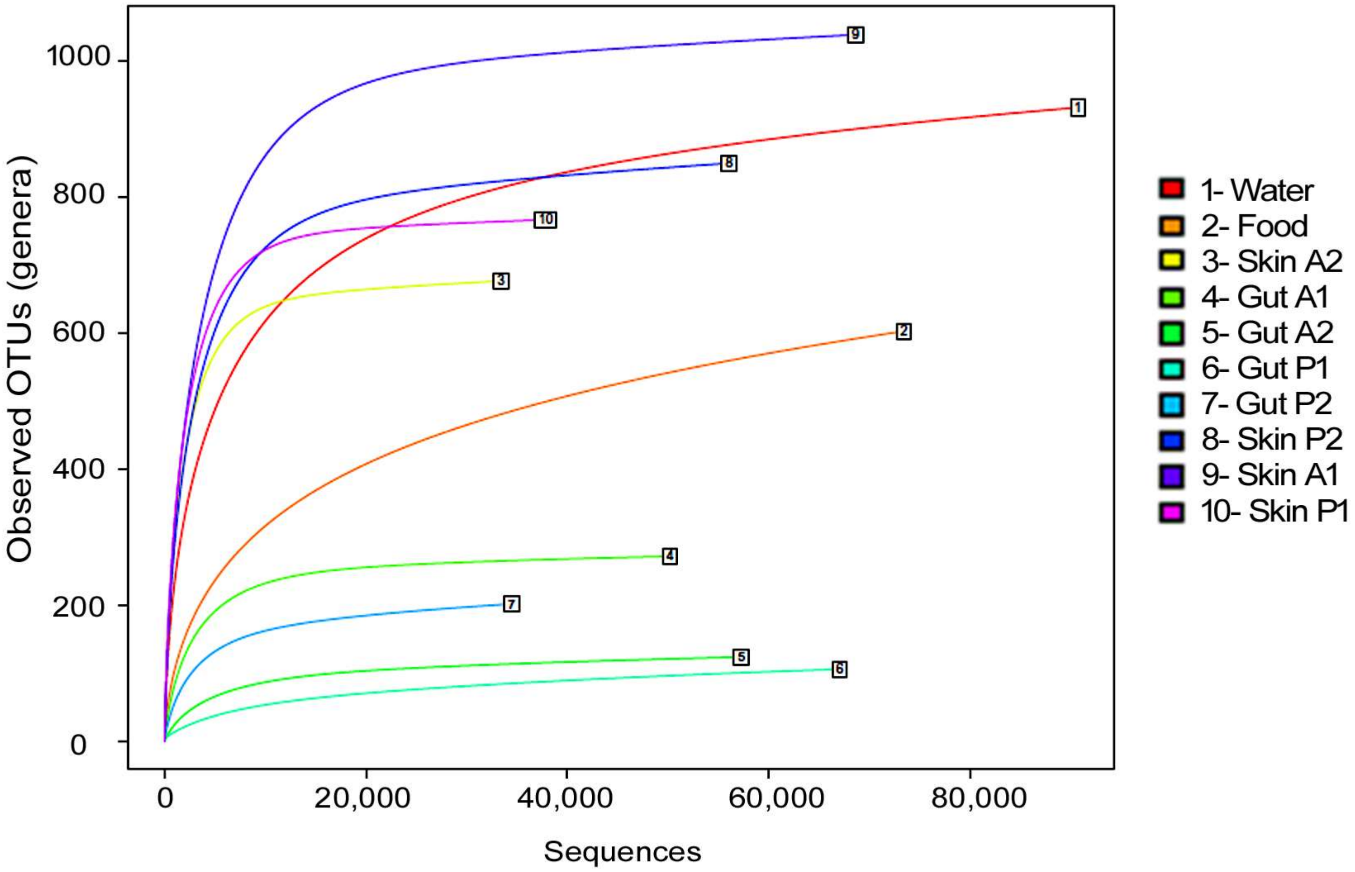

Supplementary Figure S2. Phylogenetic tree of fish agouti-signaling proteins (ASIP1/ASIP2) and Agouti-related proteins (AGRP/AGRP2) with the tetrapod homologues.
The tree was constructed with the Maximum-likelihood algorithm with PhyML (Guindon and Gascuel 2003) with 100 bootstrap replicates. Branch support values are shown for the major protein family clades. The three major clusters are identified with different colours. The new ASIP2 protein from *S. senegalensis* clusters with other ASIP2/AGRP2, confirming its identity. The accession numbers of the sequences used to build the tree are listed in Supplementary Table S10.

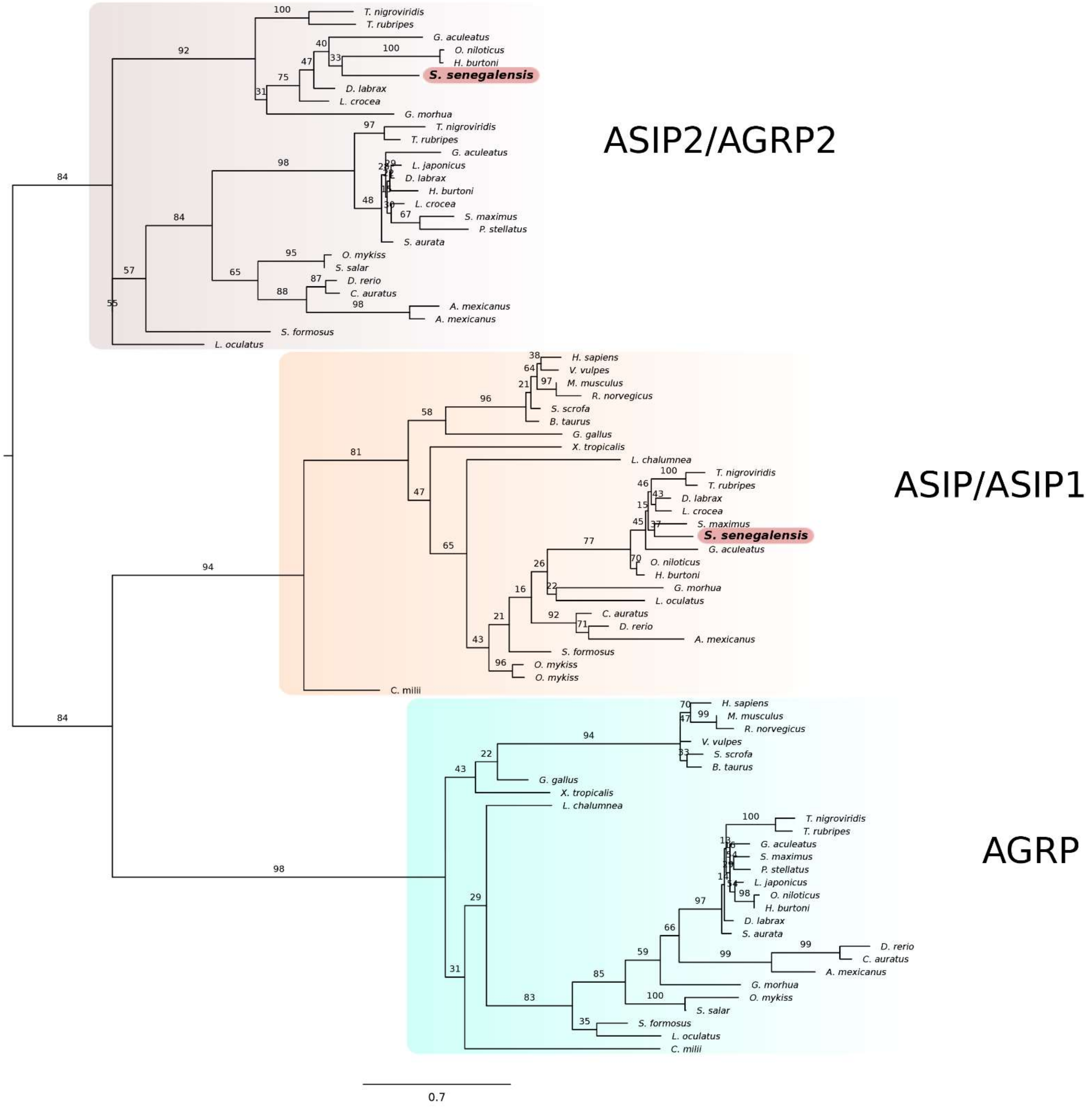

Reference:

Guindon, S., Lethiec, F., Duroux, P., & Gascuel, O. (2005). PHYML Online—a web server for fast maximum likelihood-based phylogenetic inference. Nucleic acids research, 33(suppl_2), W557-W559.
